# The microRNA-302b-inhibited insulin-like growth factor-binding protein 2 signaling pathway induces glioma cell apoptosis by targeting nuclear factor IA

**DOI:** 10.1371/journal.pone.0173890

**Published:** 2017-03-21

**Authors:** Chin-Cheng Lee, Peng-Hsu Chen, Kuo-Hao Ho, Chwen-Ming Shih, Chia-Hsiung Cheng, Cheng-Wei Lin, Kur-Ta Cheng, Ann-Jeng Liu, Ku-Chung Chen

**Affiliations:** 1 Department of Pathology and Laboratory Medicine, Shin Kong Wu Ho-Su Memorial Hospital, Taipei, Taiwan; 2 Graduate Institute of Medical Sciences, College of Medicine, Taipei Medical University, Taipei, Taiwan; 3 Department of Clinical Pharmacy, School of Pharmacy, Taipei Medical University, Taipei, Taiwan; 4 Department of Biochemistry and Molecular Cell Biology, School of Medicine, College of Medicine, Taipei Medical University, Taipei, Taiwan; 5 Department of Neurosurgery, Taipei City Hospital Ren-Ai Branch, Taipei, Taiwan; Swedish Neuroscience Institute, UNITED STATES

## Abstract

MicroRNAs are small noncoding RNAs that post-transcriptionally control the expression of genes involved in glioblastoma multiforme (GBM) development. Although miR-302b functions as a tumor suppressor, its role in GBM is still unclear. Therefore, this study comprehensively explored the roles of miR-302b-mediated gene networks in GBM cell death. We found that miR-302b levels were significantly higher in primary astrocytes than in GBM cell lines. miR-302b overexpression dose dependently reduced U87-MG cell viability and induced apoptosis through caspase-3 activation and poly(ADP ribose) polymerase degradation. A transcriptome microarray revealed 150 downregulated genes and 380 upregulated genes in miR-302b-overexpressing cells. Nuclear factor IA (NFIA), higher levels of which were significantly related to poor survival, was identified as a direct target gene of miR-302b and was involved in miR-302b-induced glioma cell death. Higher NFIA levels were observed in GBM cell lines and human tumor sections compared with astrocytes and non-tumor tissues, respectively. NFIA knockdown significantly enhanced apoptosis. We found high levels of insulin-like growth factor-binding protein 2 (IGFBP2), another miR-302b-downregulated gene, in patients with poor survival. We verified that NFIA binds to the IGFBP2 promoter and transcriptionally enhances IGFBP2 expression levels. We identified that NFIA-mediated IGFBP2 signaling pathways are involved in miR-302b-induced glioma cell death. The identification of a regulatory loop whereby miR-302b inhibits NFIA, leading to a decrease in expression of IGFBP-2, may provide novel directions for developing therapies to target glioblastoma tumorigenesis.

## Introduction

MicroRNAs (miRNAs) are endogenous small noncoding RNAs that posttranscriptionally control the expression of genes by binding to their target mRNAs for degradation or translational repression. Several miRNAs regulate various physiological cellular processes, including cell differentiation, proliferation, and apoptosis. Abnormal miRNA levels have been implicated in disease development, including glioblastoma multiforme (GBM) [1]. miRNA-21 (miR-21), an oncogenic miRNA, protects U87-MG cells from temozolomide-induced apoptosis [2]. Conversely, miR-128 downregulates E2F transcription factor 3a (E2F3a) in inhibiting glioblastoma proliferation [3]. However, only a basic understanding has been obtained for the function and role of miRNAs in GBM tumorigenesis. Greater efforts are required to obtain a clearer understanding of the role of miRNAs in GBM.

The nuclear factor I (NFI) family of transcription factors, including NFIA, NFIB, NFIC, and NFIX/NFID, promotes astrocyte differentiation and gliogenesis in the developing central nervous system [4]. NFIA is necessary to specify glial cell identity in ventricular zone progenitors in developing murine and avian spinal cords [5]. Recently, several studies have suggested that NFIA participates in GBM tumorigenesis. Highly expressed NFIA inhibits the expression of p53, p21, and plasminogen activator inhibitor 1 (PAI1) through transcriptional repression, resulting in GBM cell proliferation [6]. Furthermore, the antagonistic relationship between NFIA and Sox10 regulates the diversification of glial lineages and glioma subtypes [7]. However, mechanisms that regulate NFIA gene expression in GBM development are still unclear.

In addition to the insulin-like growth factor (IGF)-mediated network being involved in embryonic development and growth, its aberrant activation has been implicated in several diseases, including carcinogenesis [8]. The IGF-binding protein (IGFBP) superfamily [9], containing IGFBP1–7, exerts inhibitory effects on the bioactivities of IGFs and plays crucial roles in repressing tumorigenesis [10]. However, numerous studies have suggested that IGFBP2 contributes to carcinogenesis, particularly that of gliomas [11]. Overexpression of the IGFBP2 protein promotes glioma stem cell survival and glioma progression [12]. Exogenous IGFBP2 promotes proliferation, invasion, and chemoresistance to temozolomide in glioma cells through the integrin β1-extracellular signal-regulated kinase pathway [13]. Consequently, a comprehensive understanding of the molecular pathways regulated by IGFBP-2 gene expression in gliomagenesis may facilitate the development of glioma therapies.

The miR-302–367 cluster comprises miR-302a, miR-302b, miR-302c, miR-302d, and miR-367. Among these miRNAs, miR-302b has been reported to be an antioncogenic miRNA for some cancers [14–16]. In our previous study [17], we found that inhibition of E2F3 by miR-302b was involved in all-trans retinoic acid-induced glioma cell apoptosis. To date, no study has comprehensively analyzed the putative target genes of miR-302b and its functions in carcinogenesis inhibition. Thus, in the present study, by examining the transcriptome of miR-302b-overexpressing cells, we investigated the miR-302b-mediated gene networks involved in the inhibition of glioma cell growth. Our results demonstrated that NFIA is a direct target gene of miR-302b. Moreover, NFIA-regulated IGFBP2 signaling pathways play a critical role in the ability of miR-302b to regulate apoptosis in glioma cells.

## Materials and methods

### Chemicals and reagents

The human glioblastoma cell lines Hs-683, M059K, and U87-MG were purchased from the Bioresource Collection and Research Center (Hsinchu City, Taiwan). Primary human astrocytes were purchased from Thermo Fisher Scientific (Waltham, MA, USA). Other cell culture-related reagents were purchased from GIBCO-BRL (Grand Island, NY, USA). Anti-caspase-3, phosphorylated (p)-Akt, and Akt antibodies were purchased from Cell Signaling Technology (Danvers, MA, USA). All other antibodies were purchased from GeneTex (Hsinchu City, Taiwan). Annexin V, propidium iodide (PI), and 3-[4,5-dimethylthiazol-2-yl]-2,5-diphenyl tetrazolium bromide (MTT) were purchased from Sigma-Aldrich (St. Louis, MO, USA). Polyvinylidene difluoride (PVDF) membranes and enhanced chemiluminescence (ECL) solution were purchased from Millipore (Billerica, MA, USA). Trizol^®^ reagent, Lipofectamine 3000, and secondary antibodies were purchased from Invitrogen (Thermo Fisher Scientific). The SYBR^®^ Green PCR Master Mix, MultiScribe^™^ Reverse Transcriptase Kit, and TaqMan^®^ miR-302b and U6B assays were purchased from Applied Biosystems (Thermo Fisher Scientific). The Nano-Glo Dual Luciferase Assay System was purchased from Promega (Madison, WI, USA). The miR-302b mimic was purchased from GenePharma (Suzhou, China). The human IGFBP-2 enzyme-linked immunosorbent assay (ELISA) kit was purchased from RayBiotech, Inc. (Norcross, GA, USA). Primer sets were synthesized by Genomics BioSci & Tech (Xizhi, New Taipei City, Taiwan). NFIA short hairpin RNAs (shRNAs) were purchased from the National RNAi Core Facility (Nankang, Taiwan). Unless specified otherwise, all reagents were of analytical grade.

### Cell culture, treatments, and transfection

U87-MG cells were maintained in minimum essential Eagle’s medium (MEM). M059K cells were maintained in a 1:1 mixture of Dulbecco’s modified Eagle’s medium (D-MEM) and Ham’s F12 medium supplemented with 2.5 mM L-glutamine. HS-683 cells were maintained in D-MEM supplemented with 4 mM L-glutamine, and astrocyte cells were maintained in D-MEM containing the N-2 Supplement. All cells were cultured in 10% fetal bovine serum (Biological Industries, Cromwell, CT, USA), 100 units/mL penicillin, 100 μg/mL streptomycin, 1 mM sodium pyruvate, and 1 mM nonessential amino acids at 37°C in a 5% CO_2_ incubator. The U87-MG cell line was established from a grade IV glioma obtained from a 44-year old patient [18]. It is well-studied and easily obtained, therefore it was used for subsequent experiments. For transfection, cells were seeded in a 12-well plate at a density of 10^5^ cells/well. After 70% confluence was achieved, cells were transfected with indicated dose of the miR-302b mimic, scrambled miRNA mimic, scrambled or NFIA shRNAs, empty or NFIA-containing pcDNA3 plasmids, and pGL3 promoter reporter plasmids by using Lipofectamine 3000 (Invitrogen), according to the manufacturer’s instructions. No difference in cytotoxicity was observed with increasing concentrations of scrambled miRNA mimic (10, 25, 50, and 100 nM), scrambled shRNAs (0.5, 1, and 2 μg), or empty pcDNA3 plasmids (0.5, 1, and 2 μg) (data not shown); therefore we used 50 nM scrambled miRNA mimic, 1 μg scrambled shRNAs, and 1 μg empty pcDNA3 plasmids, respectively, as representative controls. After 24-h incubation, cells were lysed for further study.

### Cell viability assay and immunoblotting

Cell viability was assessed using the MTT assay. Cells were seeded overnight in a 96-well plate at 8 × 10^3^ cells/well, followed by transfection with various concentrations of the miR-302b mimic for another 24 h. Before the end of treatment, 0.5 mg/mL MTT was added to each well for 4 h. Supernatants were carefully aspirated, and formazan crystals were dissolved using dimethyl sulfoxide. The absorbance was measured at 550 nm on a Thermo Varioskan Flash reader.

For immunoblot analysis, cells were harvested in RIPA buffer (1% Nonidet P-40, 0.5% deoxycholate, and 0.1% sodium dodecylsulfate [SDS] in phosphate-buffered saline [PBS]) containing a protease inhibitor cocktail (Calbiochem, Billerica, MA, USA) and then centrifuged at 12,000 rpm for 10 min at 4°C. The supernatant was used as the total cell lysate. Lysates (20 μg) were denatured in 2% SDS, 10 mM dithiothreitol, 60 mM Tris-hydrochloric acid (Tris-HCl, pH 6.8), and 0.1% bromophenol blue and loaded onto 10%–15% polyacrylamide/SDS gel. Separated proteins were then transferred onto a PVDF membrane. The membrane was blocked in PBS containing 5% nonfat dry milk for 1 h at room temperature and incubated in PBS-T containing the primary antibody overnight at 4°C. The membrane was washed in PBS-T, incubated with the horseradish peroxidase-conjugated secondary antibody for 1 h at room temperature, and then again washed in PBS-T. An ECL nonradioactive detection system was used to detect the antibody—protein complexes.

### Apoptosis analysis and microarray, pathway analysis, and survival rate analysis

Apoptosis was analyzed using flow cytometry with annexin V/PI double staining to detect membrane events. In brief, after cells were transfected with the miR-302b mimic for 24 h, whole cells were collected in Hepes buffer containing 10 mM Hepes (pH 7.4), 140 mM NaCl, and 2.5 mM CaCl_2_. Subsequently, cells were stained with annexin V (2.5 μg/mL) and PI (2 ng/mL) for 20 min, followed by analysis on a flow cytometer by using CellQuest software (Becton Dickinson, San Jose, CA, USA). The four quadrants of the cytogram in the figures were used to distinguish normal (annexin V−/PI−), early apoptotic (annexin V+/PI−), late apoptotic (annexin V+/PI+), and necrotic (annexin V−/PI+) cells. Total apoptosis was calculated as the sum of early and late apoptotic cells.

For a microarray, total RNA was isolated from U87-MG cells with or without transfection with 50 nM miR-302b mimic for 24 h. Total RNA was extracted from cultured cells by using Trizol^®^, according to the manufacturer’s instructions. RNA quality was assessed by Agilent Bioanalyzer (A260/A280 ≥ 1.8, A260/A230 ≥ 1.5, and RIN ≥ 6). Gene expression profiling was performed using the Human Whole Genome OneArray TM, Version 6.1 (Phalanx Biotech Group, Hsinchu City, Taiwan). All experiments including complementary RNA (cRNA) amplification and hybridization, image scanning by using the Axon 4000 scanner (Molecular Devices, Sunnyvale, CA, USA), and statistical analysis by using Genepix software (Molecular Devices) were conducted by the Phalanx Biotech Group. The log2 (ratio) was calculated according to a pair-wise combination and error weighted average. Significantly differentially expressed (DE) gene lists were filtered using a *p* value of <0.05 and a |log2 (ratio)| of ≥1 (±2 multiples of change) as cutoffs for further analysis. Hierarchical clustering and heat map analysis were performed using CIMminer [19]. Pathway enrichment and gene function analyses were performed using DAVID Bioinformatics Resources 6.7 [20] and ingenuity pathway analysis (IPA; QIAGEN Silicon Valley, CA, USA). For survival rate analysis, GSE16011 and GSE7696 array data were analyzed using the SurvExpress platform [21]. The GSE16011 database [22] consists of transcriptome profiling obtained using Affymetrix gene arrays of 276 glioma samples of varied histology and 8 control samples while the GSE7696 database [23] consists of 80 glioblastoma specimens and 4 non-tumoral brain samples. Both were downloaded from the GEO dataset (www.ncbi.nlm.nih.gov/geo/). All probe sets were averaged per sample. The Kaplan—Meier log-rank test and Cox proportional hazard regression were used to estimate the relationship of NFIA and IGFBP2 expression with survival time. High- and low-risk groups with significant differences in survival were generated by maximizing risk groups with Kaplan—Meier log-rank tests. The high and low risk group were categorized base on the prognostic index risk score estimated by beta coefficients multiplied by gene expression values. By changing the cut-off point of risk score one patient at a time until a certain limit, a log-rank test p value was estimated. When this p-value was minimum, the cut-off point was chosen. The target genes of miR-302b were predicted using TargetScan 6.2 [24].

### RNA isolation and real-time reverse transcription-polymerase chain reaction

Cells were grown overnight to 80% confluency in serum-containing media. After transfecting with indicated dose of mimics or plasmids for 24 h, cell lysates were collected. Total RNA was extracted from cell lysates by using Trizol^®^, according to the manufacturer’s instructions. RNA quality was assessed by calculating the A260/A280 ratio. cDNA was synthesized from 1 μg of total RNA by using a random primer and the MultiScribe^™^ Reverse Transcriptase Kit. cDNA was diluted to 1:30 with polymerase chain reaction (PCR)-grade water and then stored at −20°C. S1 Table lists the specific primers for human NFIA, IGFBP2, and GAPDH for real-time PCR (qPCR). Gene expression levels were quantified using the Applied Biosystems StepOnePlus^™^ System (Thermo Fisher Scientific) with preoptimized conditions. Each PCR was performed in triplicate by using 5 μL of 2× SYBR^®^ Green PCR Master Mix, 0.2 μL of primer sets, 1 μL of cDNA, and 3.6 μL of nucleotide-free H_2_O to yield 10 μL per reaction. The relative expression levels of NFIA and IGFBP2 were normalized respectively to GAPDH by using the equation ΔCt = (Ctgene—CtGAPDH). The ΔΔCt value was calculated by ΔCt (treat) - ΔCt (control). Finally, the fold change was calculated by 2(-ΔΔCt). The mean and standard deviation of fold change were calculated.

To detect miR-302b and U6B, cDNA was synthesized from 5ng/μl total RNA by using TaqMan^®^ MicroRNA Assays (Applied Biosystems) and then stored at −20°C. MicroRNA expression levels were quantified using the Applied Biosystems StepOnePlus^™^ System (Thermo Fisher Scientific) with preoptimized conditions. Each PCR was performed in triplicate by using 5 μL of 2×TaqMan^®^ Universal PCR Master Mix, 0.5 μL of primer sets, and 4.5 μL of cDNA to yield 10 μL per reaction. The relative expression levels of miR-302b were normalized respectively to U6B by using the equation ΔCt = (CtmiR-302b –CtU6b). The ΔΔCt value was calculated by ΔCt (treat) - ΔCt (control). Finally, the fold change was calculated by 2(-ΔΔCt). The mean and standard deviation of fold change were calculated.

### Construction of the NFIA 3′UTR reporter plasmid and mutagenesis

PCR was performed using sets of primers specific for NFIA 3′UTR, of which the forward primer contained the *Xho*I site, and the reverse primer contained the *Xba*I site. S1 Table lists the PCR primers. cDNA extracted from U87-MG cells was used as a template. Three individual 500-bp PCR products containing different putative miR-302b binding sites were digested with *Xho*I and *Xba*I and cloned downstream of the luciferase gene in the pMIRGLO-REPORT luciferase vector (Promega). This vector was sequenced and named pMIRGLO-NFIA-3-1, -2, and -3. Site-directed mutagenesis of the miR-302b target site in NFIA 3U-3 was performed using the QuikChange^®^ Site-Directed Mutagenesis Kit (Stratagene, Heidelberg, Germany), and the vector was named pMIRGLO-NFIA-3′UTR-mutant. For reporter assays, cells were transfected transiently with the wild-type or mutant reporter plasmids and the miR-302b mimic by using Lipofectamine 3000 (Invitrogen). The reporter assay was performed 24 h after transfection by using the Luciferase Assay System (Promega). The dual Renilla luciferase value was used as an internal control.

### Immunohistochemistry and measurement of extracellular IGFBP2 levels using ELISA

Three formalin-fixed, paraffin-embedded surgical samples of GBM patients were obtained from the Department of Pathology and Laboratory Medicine, Shin Kong Wu Ho-Su Memorial Hospital. For immunohistochemistry (IHC), 5-μm paraffin-embedded sections were mounted on poly-L-lysine-coated slides and baked at 60°C overnight. After dewaxing and hydration, the sections were heated in a microwave oven for 10 min with 10 mmol/L sodium citrate buffer (pH 6). After blocking with 10% horse serum in PBS for 30 min, the sections were incubated overnight at 4°C with the anti-NFIA primary antibody. On the next day, the sections were incubated for 30 min in biotinylated goat anti-rabbit immunoglobulin G (IgG) (1:1000 dilution), followed by detection of immunoreactivity by using an avidin—biotin system that utilized 3, 3′-diaminobenzidine tetrahydrochloride as a chromogen. The sections were lightly counterstained with Mayer’s hematoxylin (Richard-Allan Scientific, Kalamazoo, MI, USA). Negative controls without primary antibodies were performed for all reactions (data not shown).

The extracellular IGFBP2 level was measured using the RayBio^®^ Human IGFBP-2 ELISA Kit according to the manufacturer’s instructions. Cells were seeded overnight to 80% confluency in a 6-well plate at 10^5^ cells/well with serum-containing mediums. After transfecting with indicated dose of mimics or plasmids for 24 h, the 1 ml conditioned media was collected. First, 100 μL of each standard and supernatant samples was respectively added into labeled removable 8-well strips, and the wells were covered and incubated for 2.5 h at room temperature with gentle shaking. Subsequently, 100 μL of the prepared biotinylated antibody, streptavidin solution, and TMB One-Step Substrate Reagent was successively added to the wells with interval washing, and the wells were incubated for 1 h at room temperature with gentle shaking. Finally, 50 μL of stop solution was added to the wells, and the absorbance was measured at 450 nm on a Thermo Varioskan Flash reader.

### Construction of full-length NFIA cDNA and the *IGFBP2* promoter reporter plasmid and mutagenesis

Full-length NFIA cDNA (NM_001134673.3) without a 3′UTR was generated through PCR amplification by using the primers listed in S1 Table. The following thermal profile was used for the PCR amplification of 500 ng of cDNA on a GeneAmp PCR system 9700 (Applied Biosystems): an initial denaturation step at 95°C for 5 min, followed by 40 cycles of 94°C for 1 min, 58°C for 1 min, and 72°C for 1 min, with a final extension at 72°C for 10 min. PCR products were analyzed using agarose gel electrophoresis. All PCR products were cloned into pGEM-T Easy (Promega Corporation) and sequenced. After *Bam*HI/*Eco*RI digestion, NFIA cDNA was cloned into pcDNA3.1 to generate a construct named pcDNA-NFIA.

To test the effects of NFIA on *IGFBP2* promoter activity, several reporter constructs were generated. A 2-kb fragment of the *IGFBP2* promoters was isolated using PCR with primers listed in S1 Table. After digestion of the PCR product with *Mlu*I and *Hind*III, the insert was cloned into the reporter vector pGL3 (Promega); thus, the expression vector pGL3-IGFBP2-prom was created. Site-directed mutagenesis of NFIA-binding sites in the *IGFBP2* promoters was performed using the QuikChange^®^ Site-Directed Mutagenesis Kit (Stratagene), and the vector was named pGL3-IGFBP2-prom-MUT, in which pGL3-IGFBP2-prom was used as a template. For reporter assays, cells were transiently cotransfected with different sizes of the wild-type or mutant reporter plasmids and NFIA plasmids or the miR-302b mimic by using Lipofectamine 3000 (Invitrogen). Additionally, cells were cotransfected with pNL1.1-TK plasmids, which served as the internal control. The reporter assay was performed 24 h after transfection by using the Nano-Glo Dual Luciferase Assay System (Promega).

### Chromatin immunoprecipitation assay

Chromatin immunoprecipitation (ChIP) assays were performed according to the manufacturer’s instructions (EZ-ChIP^™^, Millipore). Briefly, 10^6^ cells with or without transfection with NFIA plasmids and the miR-302b mimic were fixed with 1% formaldehyde, washed with cold PBS, and lysed in buffer. Nuclei were sonicated to shear the DNA, and lysates were pelleted and precleared. Protein—DNA complexes were incubated overnight with 1 μg of antibodies and then incubated with protein G beads, followed by elution in 1% SDS/0.1 M NaHCO_3_ and cross-link reversal at 65°C. DNA recovered from samples containing the NFIA antibody was compared with the negative control (mouse IgG) and the positive control (anti-RNA-Pol antibody) provided by the manufacturer. After recovery, DNA was analyzed using PCR. S1 Table lists the PCR primers.

### Statistical analysis

All data are presented as the mean ± SD. Significant differences among groups were determined using unpaired t' test and one way ANOVA with Tukey—Kramer multiple comparison test. A value of *p* of < 0.05 was taken as an indication of statistical significance. All figures shown in this article were obtained from at least three independent experiments with similar results.

## Results

### miR-302b induced glioma cell death through apoptosis

**Fig 1:**
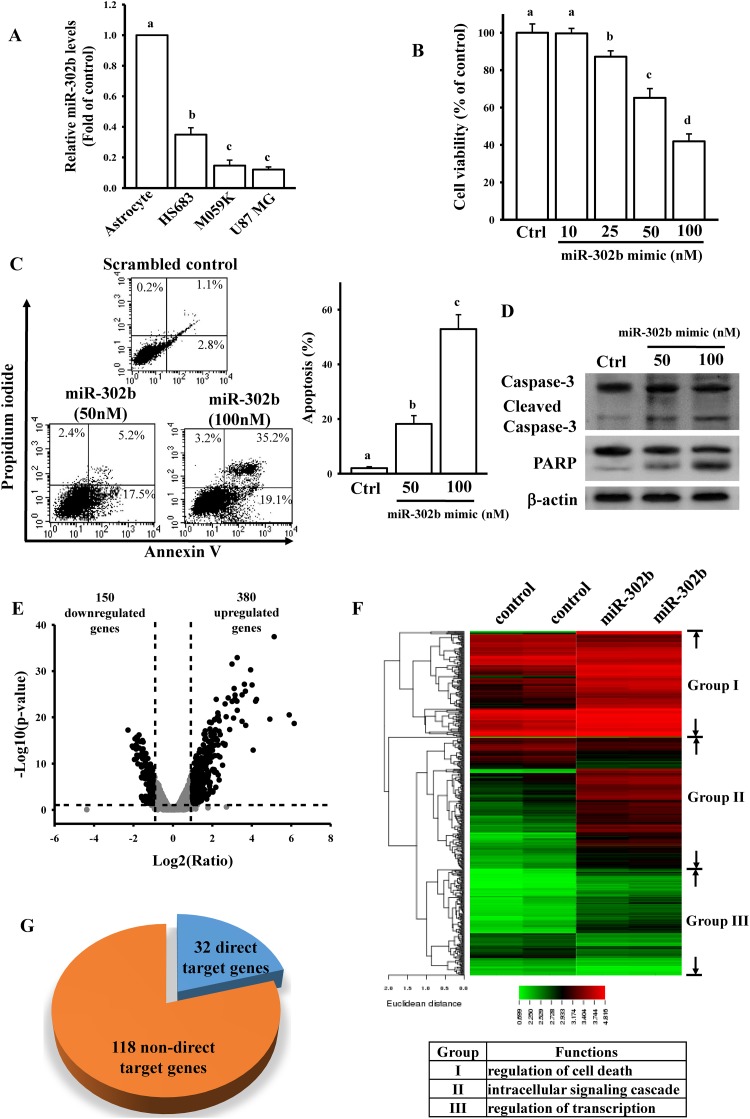
Effects of miR-302b on mediating glioma cell death. (A) Detection of endogenous miR-302b levels in normal human astrocytes and the three glioma cell lines of HS683, M059K, and U87-MG. Cells were grown overnight to 80% confluency in serum-containing media. After spent media, total lysates were used to extract the total RNAs for measuring relative miR-302b and U6b levels by real-time PCR. Data are expressed as the mean ± SD of three independent experiments. After analyzing by one way ANOVA with Tukey—Kramer multiple comparison test, different letters above bars denote samples that were significantly different (*p* < 0.05) compared with astrocyte. (B) Effects of miR-302b overexpression on cell viability. Overnight culture U87-MG cells were transfected with different doses of the miR-302b mimic for 24 h. The scrambled miRNA mimic (50 nM) was used as a control (Ctrl). Cell viability was measured using the MTT assay. Data are expressed as the mean ± SD of three independent experiments. After analyzing by one way ANOVA with Tukey—Kramer multiple comparison test, different letters above bars denote samples that were significantly different (*p* < 0.05) compared with control. miR-302b overexpression enhanced cell apoptosis (C), caspase-3 activation, and PARP degradation (D). After overnight culture cells were transfected with the indicated dose of the miR-302b mimic and 50 nM scrambled miRNA mimic (Ctrl) for 24 h, the apoptosis ratio and caspase-3 and PARP levels were measured using flow cytometry with annexin V/PI double staining and immunoblotting assays. The right panel in (C) shows quantitative results of three independent experiments. Data are expressed as the mean ± SD of three independent experiments. After analyzing by one way ANOVA with Tukey—Kramer multiple comparison test, different letters above bars denote samples that were significantly different (*p* < 0.05) compared with control. Volcano plot (E) and heat map of hierarchical gene clustering (F) demonstrating miR-302b-regulated transcriptome profiles. After overnight culture U87-MG cells were transfected with 50 nM miR-302b mimic and 50 nM scrambled miRNA mimic (control) for 24 h, total RNA was extracted for the microarray analysis. For all array analyses, a *p* value of <0.05 and a ± 1 multiple of change cutoff were applied. Volcano plots show multiples of change (log_2_ ratio) and probability (-log_10_ *p* values) for individual mRNA from the microarray assay. Significantly upregulated and downregulated mRNAs are represented by black dots. The heat map depicts the 530 mRNAs that were differentially expressed between the miR-302b subsets. A color was assigned to each mRNA on the basis of its relative expression across samples. The network of overlapping canonical pathways was analyzed using DAVID Bioinformatics Resources 6.7 according to the 150 downregulated and 380 upregulated molecule-related pathways in miR-302b-overexpressing cells. (G) Identification of direct and non-direct target genes of miR-302b from 150 downregulated genes. The target genes were predicted using TargetScan 6.2.

To explore the role of miR-302b in regulating glioma cell death, we compared the endogenous miR-302b levels in normal astrocytes and three glioma cell lines (Fig 1A). We found that miR-302b expression levels were lower in glioma cells than in primary astrocytes. To test the cytotoxic effects of miR-302b on U87-MG cells, cells were transfected with different doses of the miR-302b mimic for 24 h (Fig 1B). We found that miR-302b induced U87-MG cell death in a dose-dependent manner. Transfection with 50 nM miR-302b mimic for 24 h reduced U87-MG cell viability by 41% in comparison with transfection with the scramble control. To investigate the type of cell death induced by miR-302b, cells transfected with different dose of miR-302b mimic and 50 nM scramble control were stained with annexin V-FITC and PI and analyzed using flow cytometry. As shown in Fig 1C, miR-302b promoted U87-MG cell death through apoptosis. miR-302b also enhanced the apoptosis ratio in a dose-dependent manner. Furthermore, caspase-3 activation and poly(ADP ribose) polymerase (PARP) degradation were detected in miR-302b-transfected U87-MG cells (Fig 1D). This observation suggests that miR-302b significantly promotes glioma cell apoptosis.

### miR-302b-mediated transcriptome in glioma cells

To explore the effects of miR-302b on glioma apoptosis, we generated a transcriptome microarray of cells transfected with 50 nM miR-302b mimic and 50 nM scramble control for 24 h. We selected significantly differentially expressed genes by using a log2 (ratio) of |≥ 1| and a *p* value of < 0.05 as cutoffs. We found that miR-302b overexpression upregulated the expression of 380 genes and downregulated the expression of 150 genes (Fig 1E and S1 Array Data). In hierarchical clustering analysis, these 530 genes were divided into three clusters (Fig 1F). The molecular and cellular functions of these three clusters included the regulation of cell proliferation, death, and cellular movement according to DAVID Bioinformatics Resources 6.7 (inset in Fig 1F) and IPA (S2 Table). Target gene prediction by using TargetScan revealed that among 150 downregulated genes, 32 genes are putative direct target genes of miR-302b (Fig 1G and Table 1). Our results collectively suggest that miR-302b-mediated gene networks are highly associated with glioma U87-MG cell death.

**Table 1:** The 32 putative miR-302b target genes.

Gene symbol	log2 (Ratio)	*p*-value (Differentially expressed)	Average Normalized Intensity	
			control	miR-302
SCN2A	-1.83	1.4376E-12	208.77	85.50
SHROOM2	-1.82	1.91273E-14	958.52	406.94
SEC16B	-1.73	2.56869E-12	475.71	215.96
METTL7A	-1.72	6.61307E-17	2542.44	1123.00
FAM46C	-1.61	1.33176E-12	581.23	287.50
C3AR1	-1.58	0.002687348	656.38	426.39
MVK	-1.51	2.22057E-10	524.15	277.52
MXD3	-1.51	6.49841E-14	809.19	426.99
OLFML2A	-1.48	2.0447E-12	1507.20	797.97
MYLK	-1.45	2.92587E-10	12881.62	6245.91
NFIA	-1.41	1.81234E-07	236.61	130.00
HTR2A	-1.33	0.039284747	176.85	99.00
KLHL3	-1.30	5.56009E-08	435.84	266.53
PRSS23	-1.28	8.01986E-10	1804.18	1088.25
MRGPRF	-1.24	2.80063E-06	2698.60	1620.40
PRUNE2	-1.18	9.8951E-06	245.89	161.02
MEF2C	-1.17	1.47851E-05	122.91	77.00
TGFBR2	-1.16	0.000129502	1170.04	777.59
TRIM2	-1.15	1.01169E-09	2140.20	1402.42
DHCR7	-1.14	1.91506E-08	6138.34	3834.95
CLCN4	-1.09	0.001194219	403.70	282.48
PRRT3	-1.08	6.08257E-09	1319.37	923.99
FANCD2	-1.07	1.02895E-05	457.49	330.52
QPRT	-1.05	2.55731E-07	7759.98	5059.73
PHKB	-1.05	1.43776E-07	357.84	262.49
COL5A1	-1.05	2.4592E-08	3680.59	2507.07
SDC1	-1.04	1.16296E-07	17256.05	10960.50
PCLO	-1.04	2.33602E-05	167.17	118.00
PKIB	-1.03	1.1431E-06	3362.71	2321.64
FBLIM1	-1.03	0.000485279	548.29	397.96
ETV1	-1.03	2.5444E-09	3446.82	2392.92
RNF157	-1.02	7.74002E-08	778.37	574.50

### Identification of NFIA as a target gene of miR-302b

**Fig 2:**
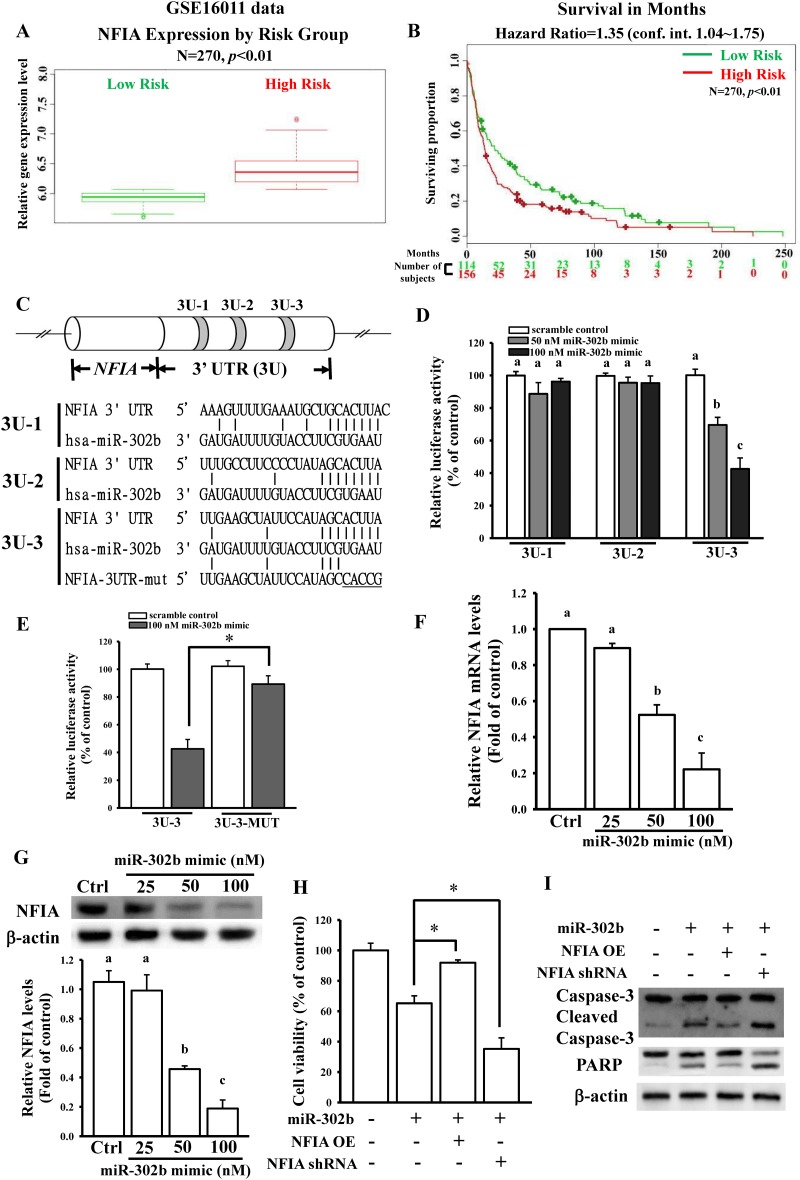
Effects of the miR-302b target gene of NFIA on regulating glioma cell death. (A) Relative NFIA expression levels according to GBM patient risk group based on GSE16011 profiling. Higher risk patients had higher NFIA expression, denoted by red symbols. Conversely, lower NFIA expression was associated with higher survival, denoted in green. (B) Kaplan—Meier analysis of patient survival data from GSE16011 profiling. The *x*-axis values indicate the number of patients per group. The red curves indicate high-risk patients. The green curves indicate low-risk patients. The plus sign indicates censored observations. The *p* values were calculated using the log-rank test. (C) Schematic diagram of potential miR-302b-targeted sites in the NFIA 3′UTR. Three putative sites were predicted and named 3U-1, -2, and -3. (D and E) Effects of miR-302b on NFIA 3′UTR luciferase activity. To test the effect of miR-302b, overnight culture cells were cotransfected with different doses of the miR-302b mimic or 50 nM scrambled miRNA mimic (control) and 500 ng of the pmiRGlo-NFIA 3′ UTR or mutant 3′UTR. Luciferase activity was measured in these cells 24 h after transfection. Effects of miR-302b overexpression on NFIA mRNA (F) and protein (G) expression. After overnight culture cells were transfected with the indicated dose of the miR-302b mimic and 50 nM scrambled miRNA mimic (Ctrl) for 24 h, the relative mRNA and protein levels of NFIA were analyzed using real-time PCR and immunoblotting assays. The lower panel in (G) shows quantitative results of three independent experiments of immunoblotting assays. Overexpression and knockdown of NFIA influenced miR-302b-mediated cell viability (H) and apoptotic markers (I). After overnight culture cells were cotransfected with 50 nM miR-302b mimic and 1 μg of the pcDNA3-NFIA OE plasmid or shRNA for 24 h, the cell viability and apoptotic markers were measured using the MTT assay and immunoblotting assays with anti-poly(ADP ribose) polymerase and caspase-3 antibodies. The scrambled miRNA mimic (50 nM), empty pcDNA3 vectors (1 μg), and scrambled shRNA (1 μg) were used as controls. Data are expressed as the mean ± SD of three independent experiments. After analyzing by one way ANOVA with Tukey—Kramer multiple comparison test, different letters above bars denote samples that were significantly different (*p* < 0.05) compared with control. *p < 0.05 by unpaired Student’s *t* test.

To further identify the direct target gene of miR-302b from 32 downregulated genes, survival rate analysis was conducted using the GSE16011 database. As shown in Fig 2A and 2B and S1 Fig, the high expression of eight genes, NFIA, C3AR1, CLCN4, COL5A1, DHCR7, FANCD2, FBLIM1, and PHKB, was statistically associated with poor survival. Because a previous study suggested that NFIA promotes glioblastoma growth [6], we focused on the effects of miR-302b on NFIA gene expression. Furthermore, survival rate analysis was conducted using another database (GSE7696), and high NFIA expression was associated with poor survival (S2A and S2B Fig). In hierarchical clustering analysis, NFIA function is classified into cluster III, which is involved in the regulation of transcription (Fig 1F and S2C Fig). Target gene prediction by using TargetScan revealed that miR-302b binds to three sites in the NFIA 3′UTR, named 3U-1, -2, and -3 (Fig 2C). Furthermore, to confirm that NFIA is a direct target gene of miR-302b, three NFIA 3′UTR regions containing a miR-302b-binding site were cloned into the pmiRGlo-reporter plasmids. As shown in Fig 2D, the miR-302b mimic significantly reduced the luciferase activity of only 3U-3 in a dose-dependent manner. To validate whether miR-302b influences NFIA expression levels by binding to the 3′UTR, five nucleotides in the critical binding region of the NFIA 3′UTR were mutated using site-directed mutagenesis (Fig 2C). This procedure reduced or abolished miR-302b binding to NFIA. As shown in Fig 2E, the miR-302b mimic did not affect luciferase activity after mutation of the miR-302b-targeted site. We also directly tested the effect of miR-302b on NFIA expression and found that transient transfection of U87-MG cells with the miR-302b mimic significantly and dose dependently reduced NFIA mRNA and protein levels, as measured through RT-qPCR (Fig 2F) and immunoblotting (Fig 2G). To determine whether NFIA is involved in miR-302b-mediated cell death, we measured NFIA levels after transfection with NFIA overexpression (NFIA OE) plasmids, NFIA shRNA, or the scramble control (S2D Fig). As shown in Fig 2H and 2I, overexpression or knockdown of NFIA significantly influenced miR-302b-regulated cell viability, caspase-3 activation, and PARP degradation. The results collectively demonstrate that miR-302b-inhibited NFIA expression results in U87-MG cell apoptosis.

### Effects of NFIA on glioma growth

**Fig 3:**
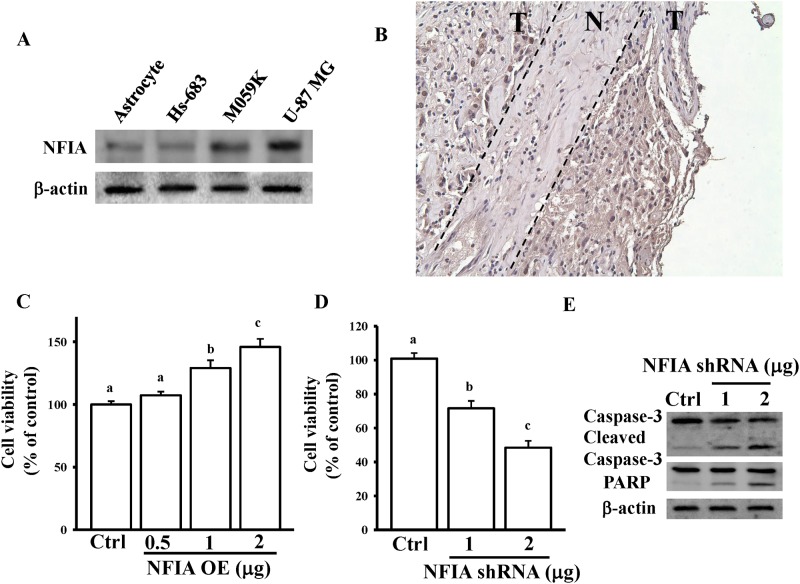
Effects of NFIA on mediating glioma cell growth. (A) Detection of endogenous NFIA protein levels in normal human astrocytes and three glioma cell lines. Cells were grown overnight to 80% confluency in serum-containing media. After spent media, total lysates were used to extract the total proteins for measuring NFIA and β-actin levels by immunoblotting assays. (B) IHC detection in formalin-fixed, paraffin-embedded surgical samples showed variations in NFIA expression in tumor tissues (T) compared with normal tissues (N). (C) NFIA overexpression enhanced U87-MG cell growth. Knockdown of NFIA reduced cell viability (D) and enhanced caspase-3 activation and PARP degradation (E). After overnight culture cells were transfected with the indicated dose of the NFIA OE plasmid or NFIA shRNA for 24 h, cell viability and caspase-3 and PARP levels were measured using the MTT assay and immunoblotting assays. The empty pcDNA3 vectors and scrambled shRNA (1 μg) were used as controls. Data are expressed as the mean ± SD of three independent experiments. After analyzing by one way ANOVA with Tukey—Kramer multiple comparison test, different letters above bars denote samples that were significantly different (*p* < 0.05) compared with control.

To investigate the role of NFIA in glioma growth, we compared the endogenous protein levels of NFIA in normal astrocytes and three glioma cell lines (Fig 3A). High protein levels of NFIA were found in glioma cells, particularly U87-MG cells. Furthermore, IHC staining of paraffin-embedded human glioma tissue sections with the anti-NFIA antibody revealed more intense staining in tumor tissues (T) compared with non-tumor tissues (N) (Fig 3B). Regarding the cytotoxic effects of NFIA, NFIA overexpression dose dependently enhanced U87-MG cell viability (Fig 3C). By contrast, NFIA knockdown significantly reduced cell viability (Fig 3D), caspase-3 activation, and PARP degradation (Fig 3E). These results suggest that abnormal expression levels of NFIA influence glioma pathogenesis.

### Identification of the NFIA-directly mediated genes in miR-302b-downregulated gene profile

**Fig 4:**
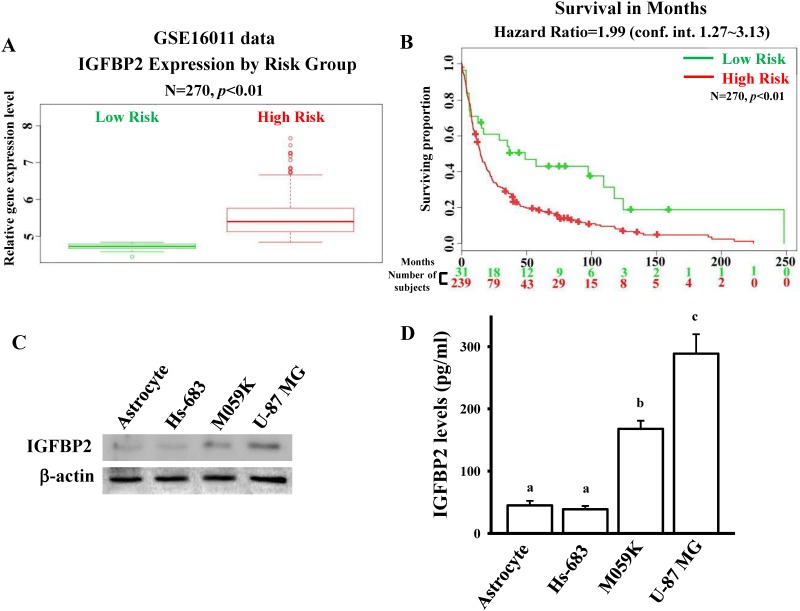
IGFBP2 is overexpressed in glioma. (A) Relative IGFBP2 expression levels according to GBM patient risk group based on GSE16011 profiling. Higher risk patients had higher IGFBP2 expression, denoted by red symbols. Conversely, lower IGFBP2 expression was associated with higher survival, denoted in green. (B) Kaplan—Meier analysis of patient survival data from GSE16011 profiling. The *x*-axis values indicate the number of patients per group. The red curves indicate high-risk patients. The green curves indicate low-risk patients. The plus sign indicates censored observations. The *p* values were calculated using the log-rank test. Detection of endogenous IGFBP2 protein levels in normal human astrocytes and three glioma cell lines by using immunoblotting assays (C) and ELISA assays (D). Cells were grown overnight to 80% confluency in serum-containing media. The total lysates and conditioned media were respectively used to measure intracellular and extracellular IGFBP2 protein levels by immunoblotting assays and ELISA assays according to the manufacturer’s instructions in method section. Data are expressed as the mean ± SD of three independent experiments. After analyzing by one way ANOVA with Tukey—Kramer multiple comparison test, different letters above bars denote samples that were significantly different (*p* < 0.05) compared with astrocyte.

Since NFIA is a transcription factor, it can bind to gene promoters, resulting in influencing gene expressions. Next, we explored the NFIA—directly and transcriptionally regulated genes that co-existed in 118 miR-302b-downregulated genes from array data, and their 3’ UTR regions did not contain miR-302b target sites. Survival rate analysis was conducted using the GSE16011 database (Fig 4A and 4B and S3 Table). Similar to NFIA, high expression of 34 genes was statistically associated with poor survival (S3 Fig). Because IGFBP2 is a well-established tumor-promoting factor in glioma [11], we investigated the role of NFIA in the regulation of IGFBP2 gene expression in glioma growth. We compared the endogenous protein levels of IGFBP2 in normal astrocytes and three glioma cell lines (Fig 4C and 4D). Higher protein levels of IGFBP2 from intracellular components and conditioned media were found in glioma cells than in normal astrocytes.

### NFIA transcriptionally regulates IGFBP2 and related downstream signaling

**Fig 5:**
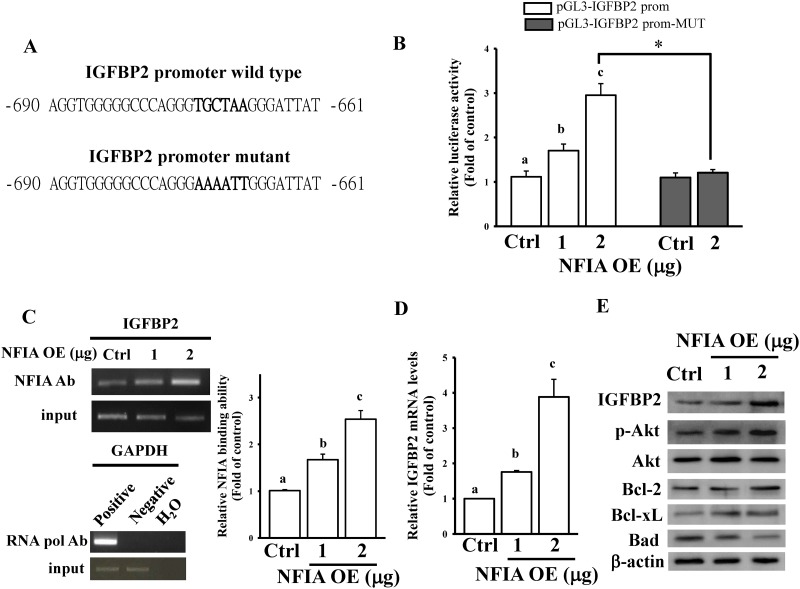
NFIA regulates the IGFBP2-mediated signaling pathway. (A) Schematic diagram of the potential NFIA-binding site in the IGFBP2 promoter region. (B) NFIA dose dependently enhanced IGFBP2 promoter activity. Mutation of the NFIA-binding site abolished NFIA-regulated IGFBP2 promoter activity. After overnight culture U87-MG cells were cotransfected with the indicated dose of NFIA OE plasmids and 500 ng pGL3-IGFBP2 prom or pGL3-IGFBP2 prom-MUT for 24 h, luciferase activity was measured. Cells were also cotransfected with pNL1.1.TK[*Nluc*/TK] plasmids (5 ng), and the NanoLucR luciferase value was used as an internal control. The empty pcDNA3 vectors (2 μg) were used as controls (Ctrl). (C) ChIP revealed that NFIA could bind to the IGFBP2 promoter. The procedure of the ChIP assay is described in the Materials and methods section. Protein—DNA complexes were incubated with anti-RNA pol or control mouse IgG antibodies, which served as positive and negative controls, respectively. The input DNA represents one-fifth of the starting material. The right panel shows the quantitative results corresponding to the data in the left panel according to densitometry. Data are expressed as the mean ± SD of three independent experiments. After analyzing by one way ANOVA with Tukey—Kramer multiple comparison test, different letters above bars denote samples that were significantly different (*p* < 0.05) compared with control. *p < 0.05 by unpaired Student’s *t* test. NFIA overexpression enhanced IGFBP2 mRNA (D) and protein levels (E). After overnight culture U87-MG cells were transfected with the indicated dose of NFIA OE plasmids or 2 μg of empty pcDNA3 vectors (Ctrl) for 24 h, relative mRNA and protein levels of IGFBP2 were measured using real-time PCR and immunoblotting assays. Data are expressed as the mean ± SD of three independent experiments. After analyzing by one way ANOVA with Tukey—Kramer multiple comparison test, different letters above bars denote samples that were significantly different (*p* < 0.05) compared with control.

Using JASPAR software [25], a putative NFIA binding site was predicted in the IGFBP2 promoter region (between -669 and -674, Fig 5A). To investigate whether NFIA binds to the IGFBP2 promoter, the 2000-bp upstream region of the IGFBP2 promoter was cloned into the pGL3-luciferase reporter vector. IGFBP2 promoter activity dose dependently increased after NFIA overexpression in U87-MG cells (Fig 5B). This observation suggests that NFIA directly mediates IGFBP2 gene expression through transcriptional regulation. Six nucleotides of the putative NFIA-binding site in the IGFBP2 promoter were mutated. Consequently, we found that NFIA exerted the strongest inhibitory effect on IGFBP2 promoter activity (Fig 5B). Using the ChIP assay, we found that NFIA can dose dependently bind to the IGFBP2 promoter (Fig 5C). Furthermore, the mRNA and protein levels of IGFBP2 significantly increased after NFIA overexpression in U87-MG cells (Fig 5D and 5E). Because PI3K/AKT-mediated Bcl-2 family proteins are crucial downstream regulators of IGF signaling, we tested the effects of NFIA on IGFBP2-related downstream signaling [26]. NFIA overexpression increased AKT phosphorylation, enhanced Bcl-2/Bcl-xL levels, and reduced Bad expression in U87-MG cells (Fig 5E). Consequently, our data suggest that NFIA enhances the IGFBP2 signaling pathway, resulting in glioma tumorigenesis.

### miR-302b inhibited IGFBP2 signaling through NFIA regulation

**Fig 6:**
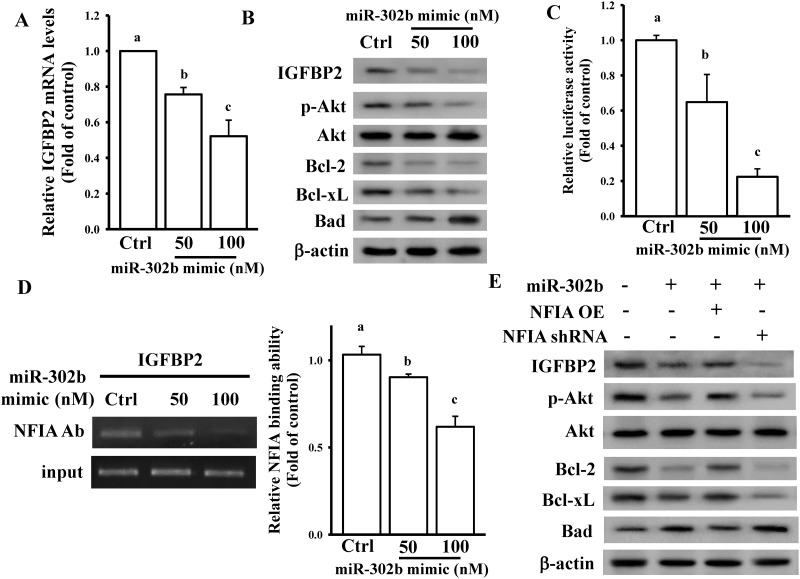
miR-302b inhibited the IGFBP2-mediated signaling pathway through NFIA regulation. Overexpression of the miR-302b mimic reduced IGFBP2 mRNA (A) and protein levels (B). After overnight culture U87-MG cells were transfected with the indicated dose of the miR-302b mimic or 50 nM scrambled miRNA mimic (Ctrl) for 24 h, relative mRNA and protein levels of IGFBP2 were measured using real-time PCR and immunoblotting assays. Data are expressed as the mean ± SD of three independent experiments. After analyzing by one way ANOVA with Tukey—Kramer multiple comparison test, different letters above bars denote samples that were significantly different (*p* < 0.05) compared with control. (C) miR-302b dose dependently reduced IGFBP2 promoter activity. After overnight culture U87-MG cells were cotransfected with the indicated dose of the miR-302b mimic, 50 nM scrambled miRNA mimic, and 500 ng of pGL3-IGFBP2 prom for 24 h, luciferase activity was measured. Cells were also cotransfected with pNL1.1.TK[*Nluc*/TK] plasmids (5 ng), and the NanoLucR luciferase value was used as an internal control. (D) ChIP revealed that miR-302b could inhibit the binding of NFIA to the IGFBP2 promoter. Data are expressed as the mean ± SD of three independent experiments. After analyzing by one way ANOVA with Tukey—Kramer multiple comparison test, different letters above bars denote samples that were significantly different (*p* < 0.05) compared with control. (E) Effects of NFIA on the miR-302b-inhibited IGFBP2 signaling pathway. After overnight culture U87-MG cells were cotransfected with 50 nM miR-302b mimic, 50 nM scrambled miRNA mimic, 1 μg of NFIA OE plasmids, empty pcDNA3 vectors, NFIA shRNA, or scrambled shRNA for 24 h, relative protein levels of IGFBP2 signaling downstream regulators were measured using immunoblotting assays.

Our array data (S2C Fig) revealed that IGFBP2 levels were decreased in miR-302b-overexpressing cells. To confirm our array result, we measured the IGFBP2 levels after miR-302b overexpression in U87-MG cells. As shown in Fig 6A and 6B, the mRNA and protein levels of IGFBP2 dose dependently decreased in miR-302b-overexpressing U87-MG cells. miR-302b overexpression also affected IGFBP2-related downstream signaling (Fig 6B). We also tested the effects of miR-302b on NFIA-regulated IGFBP2 promoter activity. miR-302b overexpression dose dependently reduced IGFBP2 promoter activity (Fig 6C). miR-302b also attenuated the binding of NFIA to the IGFBP2 promoter (Fig 6D). Furthermore, overexpression and knockdown of NFIA significantly influenced miR-302b-inhibited IGFBP2 and IGFBP2-related downstream signaling in U87-MG cells (Fig 6E). These results collectively demonstrate that inhibition of NFIA-regulated IGFBP2 signaling is involved in miR-302b-induced glioma cell death.

## Discussion

In this study, transcriptome profiles revealed that miR-302b-mediated gene networks were involved in regulating glioma cell apoptosis. Furthermore, analysis of molecular functions revealed that most miR-302b-affected genes were involved in regulation of glioma cell proliferation and death. NFIA, a transcription factor downregulated in miR-302b-overexpressing cells, was validated as a direct target gene of miR-302b. Inhibition of NFIA expression induced glioma cell death. Moreover, although IGFBP2, another tumor-promoting factor, was not the miR-302b target gene, its levels were decreased in miR-302b-overexpressing cells. Both NFIA and IGFBP2 levels were higher in glioma cell lines and tumor tissues and were significantly associated with a poor survival rate. Furthermore, NFIA could bind to the IGFBP2 promoter and transcriptionally enhanced IGFBP2 expression, resulting in activation of IGFBP2 downstream signaling. According to our results, we conclude that inhibition of NFIA-mediated IGFBP2 signaling plays a crucial role in miR-302b-induced glioma U87-MG cell death. A direct miRNA-targeted mechanism that guided nontargeting miRNA-inhibited signaling was verified to participate in miR-302b-mediated gene networks.

Several studies have reported the tumor suppressor role of miR-302b [15, 16, 27], and that miR-302b enhances the sensitivity of cancer cells to clinical therapeutic drugs [28–30]. However, few studies have focused on the effects of miR-302b on gliomagenesis. In the present study, we found that miR-302b overexpression significantly enhanced glioma cell death through apoptosis. We comprehensively analyzed the miR-302b-mediated gene networks by generating a transcriptome microarray and verified that NFIA is a novel direct target gene of miR-302b. In addition to NFIA, reduced E2F3 level, another target gene identified in our previous study [17], was found in the present microarray results (S1 Array Data). Both transcription factors have been reported to be involved in glioma development, suggesting that targeting these factors is a novel therapeutic strategy for glioma.

Previous studies have also suggested that miRNAs regulate NFIA gene expression. miR-29a significantly reduced esophageal carcinoma cell proliferation and migration by inhibiting NFIA [31]. In neural stem cells, NFIA inhibition by miR-153 repressed early gliogenesis [32]. In glioma cells, the miR-223/NFIA axis suppressed glial precursor proliferation and tumorigenesis [33]. Similarly, in the present study, we found that miR-302b induced glioma cell death by targeting and inhibiting NFIA expression. These results suggest that posttranscriptional regulatory mechanisms are crucial in mediating endogenous NFIA levels.

NFIA, identified as the CCAAT box element-binding transcription factor, is necessary to promote glial development and glioma tumorigenesis. Investigating NFIA-mediated downstream genes is crucial for exploring the molecular functions of NFIA. NFIA exerts the strongest positive effect on enhancing the promoter activity of glial fibrillary acidic protein, a differentiation marker for astrocytes [34]. NFIA can also form a complex with Sox9 and coregulate Apcdd1 and Mmd2 gene expression during astrogliogenesis [35]. Conversely, NFIA transcriptionally suppresses p53, p21, and PAI1 promoter activities and gene expression to promote GBM development [6]. In the present study, using the promoter assay and ChIP, we identified that NFIA could bind to the IGFBP2 promoter and enhance the activation of IGFBP2-related downstream signaling. NFIA-activated IGFBP2 expression also exhibited antagonistic effects on miR-302b-induced glioma cell apoptosis. Our findings identified the novel molecular mechanisms underlying the regulation of gliomagenesis by both miR-302b and NFIA.

Several findings have suggested the tumor-promoting role of IGFBP-2 in GBM and its association with a poor prognosis [11]. However, most studies have examined the functions and downstream signaling of IGFBP2. No study has focused on the regulatory mechanisms of the *IGFBP2* gene, particularly in glioma. According to our findings, IGFBP2 levels may be transcriptionally upregulated by NFIA overexpression and indirectly inhibited by miR-302b. Phillips et al. suggested that IGFBP2 is a viable therapeutic target for glioma [36]. Our findings provide different insights into the causes of gliomas and innovative therapeutic strategies. Moreover, in our miR-302b-mediated gene profiles, the expression levels of hundreds of genes were significantly changed. However, their functions should be studied to construct integrated miR-302b-regulated gene networks.

## Supporting information

S1 Table Primer list. (PDF)

S2 Table Molecular and cellular functions of miR-302b-influenced genes by ingenuity pathway analysis. (PDF)

S3 Table The 34 non-direct targets of miR-302b-downregulated genes. (PDF)

S1 Fig Another seven target genes of miR-302b possessed characteristics similar to those of NFIA. (PDF)

S2 Fig Validation of the effects of NFIA on patient survival in another GEO dataset profiling. (PDF)

S3 Fig Another 33 nondirect target genes of miR-302b possessed characteristics similar to those of NFIA. (PDF)

S1 Array Data Gene list from microarray analyses. (XLSX)
