# Supplementary material for: The microRNA-302b-inhibited insulin-like growth factor-binding protein 2 signaling pathway induces glioma cell apoptosis by targeting nuclear factor IA
Source: PLoS One. 2017 Mar 21;12(3):e0173890. doi: 10.1371/journal.pone.0173890 (PMC5360322; doi:10.1371/journal.pone.0173890)
Supplement: S1 Table — (PDF) [file pone.0173890.s001.pdf]

S1 Table. Primer list

Primer name	Sequence
**For promoter cloning**	
IGFBP2-Prom2000-F	ATATACGCGTTCCCAAGTTGCTGGGACTACAGGCGTG
IGFBP2-Prom2000-R	ATATAGATCTCTGGCCTGACTATGACTCCTGAAGGAG
**For promoter mutagenesis**	
IGFBP2-Prom-Mut-F	TAGGTGGGGGCCCAGGGAAAATTGGGATTATTTTAGCGGG
IGFBP2-Prom-Mut-R	CCCGCTAAAATAATCCCAATTTTCCCTGGGCCCCCACCTA
**For ChIP assay**	
IGFBP2-CHIP-F	GACCCCTGCAACTGAGTGTT
IGFBP2-CHIP-R	AACCTGGGAGCTCGTGACTA
**For NFIA gene cloning**	
NFIA-clon-F	TATTCTAGATTATCCCAGGTACCAGGACTGTGTCTGTTGAGGGATATTT
NFIA-clon-R	TATCTCGAGTTATCCCAGGTACCAGGACTGTGTCTGTTGAGGGATATTT
**For real-time PCR**	
NFIA-sybr-F	GCAGGCCCGAAAACGAAAATA
NFIA-sybr-R	TTTGCCAGAAGTCGAGATGCC
IGFBP2-sybr-F	TGCACATCCCCAACTGTGAC
IGFBP2-sybr-R	TGTAGAAGAGATGACACTCGGG
GAPDH-sybr-F	GTG AAG GTC GGA GTC AAC
GAPDH-sybr-R	GTT GAG GTC AAT GAA GGG
**For NFIA 3’ UTR cloning**	
NFIA-3U-1-F	ATTCTCGAGTTGAGCATTGAGGAGGCACATGGAGA
NFIA-3U-1-R	ATTTCTAGATAGCTCCCCTTTTTCTTGCAAGGCTGT
NFIA-3U-2-F	ATTCTCGAGGCTCCCACACCCCATTTTTCTTAGCA
NFIA-3U-2-R	ATTTCTAGAACATATCTTCTGAGCGGGCAGTT
NFIA-3U-3-F	ATTCTCGAGGTAAGGAGAGCTTCAGTGGCACCTC
NFIA-3U-3-R	ATTTCTAGATGACCAAAAAGGCAAGGATTGAA
**For 3’ UTR mutagenesis**	
NFIA-3U-MUT-F	CATTGAAGCTATTCCATAGCCACCGACTGTAGTGAATACTGTGT
NFIA-3U-MUT-R	ACACAGTATTCACTACAGTCGGTGGCTATGGAATAGCTTCAATG
