# Supplementary material for: The microRNA-302b-inhibited insulin-like growth factor-binding protein 2 signaling pathway induces glioma cell apoptosis by targeting nuclear factor IA
Source: PLoS One. 2017 Mar 21;12(3):e0173890. doi: 10.1371/journal.pone.0173890 (PMC5360322; doi:10.1371/journal.pone.0173890)
Supplement: S2 Table — (PDF) [file pone.0173890.s002.pdf]

S2 Table. Molecular and Cellular Functions of miR-302b-influenced genes by Ingenuity Pathway Analysis

Name	*p*-value	Molecule numbers
Cellular Growth and Proliferation	4.03E-20 - 2.76E-03	174
Cell Death and Survival	4.08E-20 - 2.70E-03	171
Cellular Development	3.19E-15 - 2.76E-03	138
Gene Expression	4.44E-14 - 9.19E-04	127
Cellular Movement	2.49E-13 - 2.76E-03	106
