# Supplementary material for: The microRNA-302b-inhibited insulin-like growth factor-binding protein 2 signaling pathway induces glioma cell apoptosis by targeting nuclear factor IA
Source: PLoS One. 2017 Mar 21;12(3):e0173890. doi: 10.1371/journal.pone.0173890 (PMC5360322; doi:10.1371/journal.pone.0173890)
Supplement: S3 Table — (PDF) [file pone.0173890.s003.pdf]

S3 Table. The 34 non-direct targets of miR-302b-downregulated genes

Symbol	Log2(ratio)	p-value	Description	Function
CDH18	-1.86	9.37E-15	Cadherin 18, Type 2	synaptic adhesion, axon outgrowth and guidance
LRRC17	-1.86	7.2E-14	Leucine Rich Repeat Containing 17	bone homeostasis
CXXC5	-1.51	2.1E-15	CXXC Finger Protein 5	Transcription factor
MT4	-1.44	7.99E-08	Metallothionein 4	zinc metabolism
FABP7	-1.41	4.38E-08	Fatty Acid Binding Protein 7	establishment of the radial glial fiber system
PCYT2	-1.41	9.92E-11	Phosphate Cytidylyltransferase 2, Ethanolamine	biosynthesis of the phospholipid phosphatidylethanolamine
GAMT	-1.39	2.45E-11	Guanidinoacetate N-Methyltransferase	synthesis of creatine from guanidinoaacetate and S-adenosylmethionine
CHST1	-1.37	2.96E-08	Carbohydrate (Keratan Sulfate Gal-6) Sulfotransferase 1	biosynthesis of selectin ligands
RTKN2	-1.36	1.34E-10	Rhotekin 2	lymphopoiesis
ID3	-1.29	2.34E-09	Inhibitor of DNA Binding 3, Dominant Negative Helix-Loop-Helix Protein	negatively regulates the basic helix-loop-helix transcription factors activities
PML	-1.22	1.38E-08	Promyelocytic Leukemia	association with PML-nuclear bodies
NDP	-1.18	1.71E-05	Norrie Disease (Pseudoglioma)	retinal developmental vasculogenesis
GUCY1B3	-1.17	5.64E-09	Guanylate Cyclase 1, Soluble, Beta 3	biosynthesis of the signaling molecule cGMP
HMGN3	-1.15	0.0005	High Mobility Group Nucleosomal Binding Domain 3	regulating chromatin structure
LOXL1	-1.15	2.32E-06	Lysyl Oxidase-Like 1	Active on elastin and collagen substrates
RNF112	-1.15	1.94E-07	Ring Finger Protein 112	GTPase activity
LMNB1	-1.14	1.01E-08	Lamin B1	components of the nuclear lamina
AS3MT	-1.13	7.49E-10	Arsenite Methyltransferase	methylates arsenite to form methylarsonate
ENC1	-1.12	3.53E-08	Ectodermal-Neural Cortex 1	differentiation of neural crest cells
IL11RA	-1.11	1.49E-07	Interleukin 11 Receptor, Alpha	development of craniofacial bones and teeth
FBN1	-1.09	1.4E-06	Fibrillin 1	maintenance of elastic fibers and anchoring epithelial cells

				to the interstitial matrix
CNIH3	-1.08	1.76E-05	Cornichon Family AMPA Receptor Auxiliary Protein 3	Regulates the trafficking and gating properties of AMPA-selective glutamate receptors
IDH1	-1.08	1.62E-07	Isocitrate Dehydrogenase 1 (NADP+), Soluble	third step of citric acid cycle
RDM1	-1.07	0.000519	RAD52 Motif Containing 1	resistance to the cisplatin
PODXL	-1.07	1.64E-08	Podocalyxin-Like	egulation of cell adhesion and morphology
GLI1	-1.07	8.58E-05	GLI Family Zinc Finger 1	transcriptional activator
FDFT1	-1.06	2.29E-08	Farnesyl-Diphosphate Farnesyltransferase 1	Farnesyltransferase
IGFBP2	-1.05	1.62E-05	Insulin-Like Growth Factor Binding Protein 2	regulator of somatic growth and cellular proliferation
CFI	-1.03	2.52E-07	Complement Factor I	Inactivates complement subcomponents C3b, iC3b and C4b
OGG1	-1.03	8.37E-08	8-Oxoguanine DNA Glycosylase	repair of oxidative DNA damage
ITGA3	-1.02	1.9E-07	Integrin, Alpha 3 (Antigen CD49C, Alpha 3 Subunit Of VLA-3 Receptor)	cell surface adhesion receptor
TTLL1	-1.02	4.34E-07	Tubulin Tyrosine Ligase-Like Family Member 1	Catalytic subunit of the neuronal tubulin polyglutamylase complex
GNG2	-1.02	0.000574	Guanine Nucleotide Binding Protein (G Protein), Gamma 2	modulator in various transmembrane signaling systems
SNAP25	-1.01	1.4E-07	Podocalyxin-Like	molecular regulation of neurotransmitter release
