# Supplementary material for: The microRNA-302b-inhibited insulin-like growth factor-binding protein 2 signaling pathway induces glioma cell apoptosis by targeting nuclear factor IA
Source: PLoS One. 2017 Mar 21;12(3):e0173890. doi: 10.1371/journal.pone.0173890 (PMC5360322; doi:10.1371/journal.pone.0173890)
Supplement: S1 Fig — (PDF) [file pone.0173890.s004.pdf]

S1 Fig

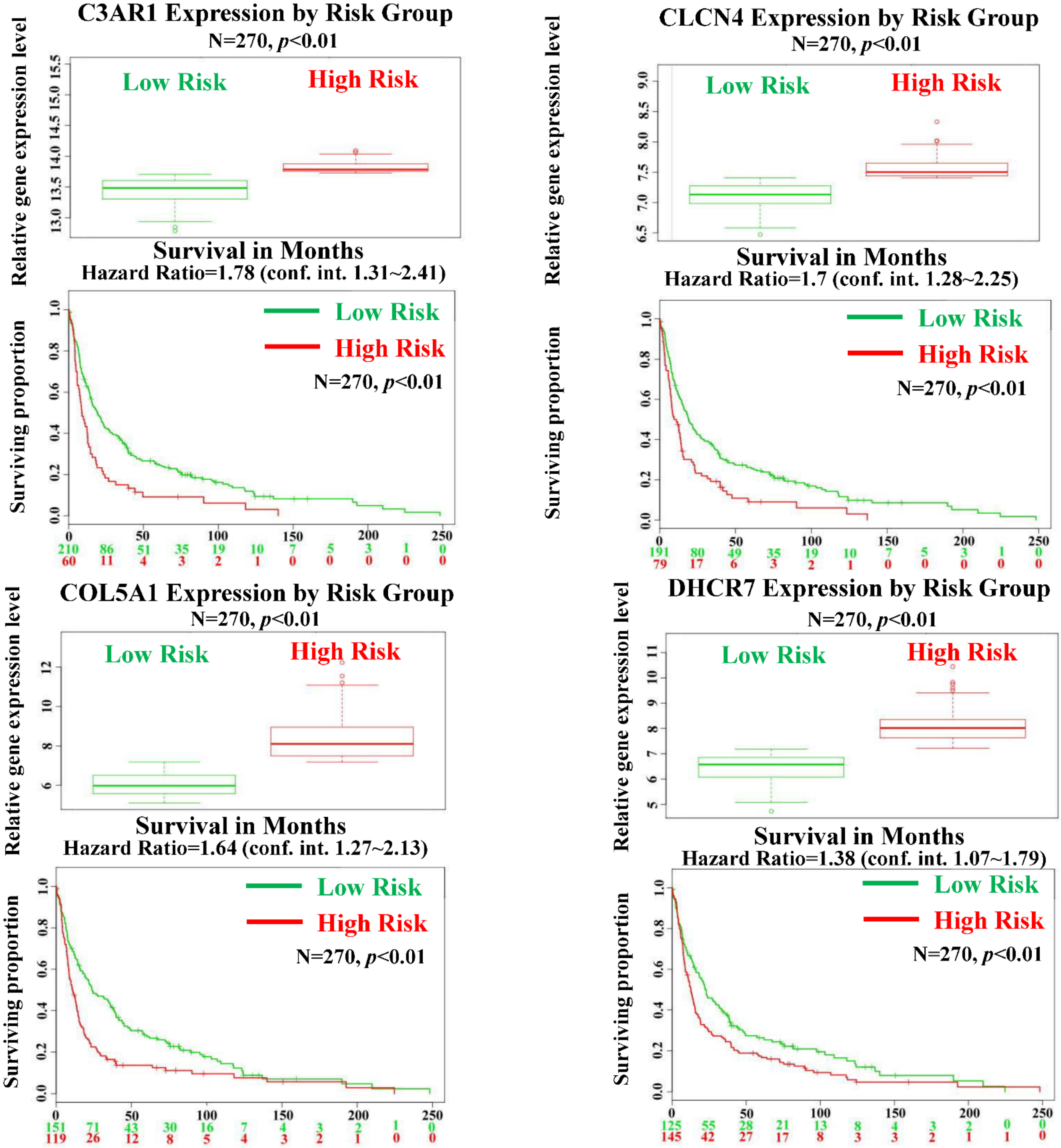
C3AR1 Expression by Risk Group
N=270, p<0.01
Low Risk
High Risk
Relative gene expression level
Survival in Months
Hazard Ratio=1.78 (conf. int. 1.31~2.41)
Surviving proportion
N=270, p<0.01
CLCN4 Expression by Risk Group
N=270, p<0.01
Survival in Months
Hazard Ratio=1.7 (conf. int. 1.28~2.25)
COL5A1 Expression by Risk Group
N=270, p<0.01
Survival in Months
Hazard Ratio=1.64 (conf. int. 1.27~2.13)
DHCR7 Expression by Risk Group
N=270, p<0.01
Survival in Months
Hazard Ratio=1.38 (conf. int. 1.07~1.79)

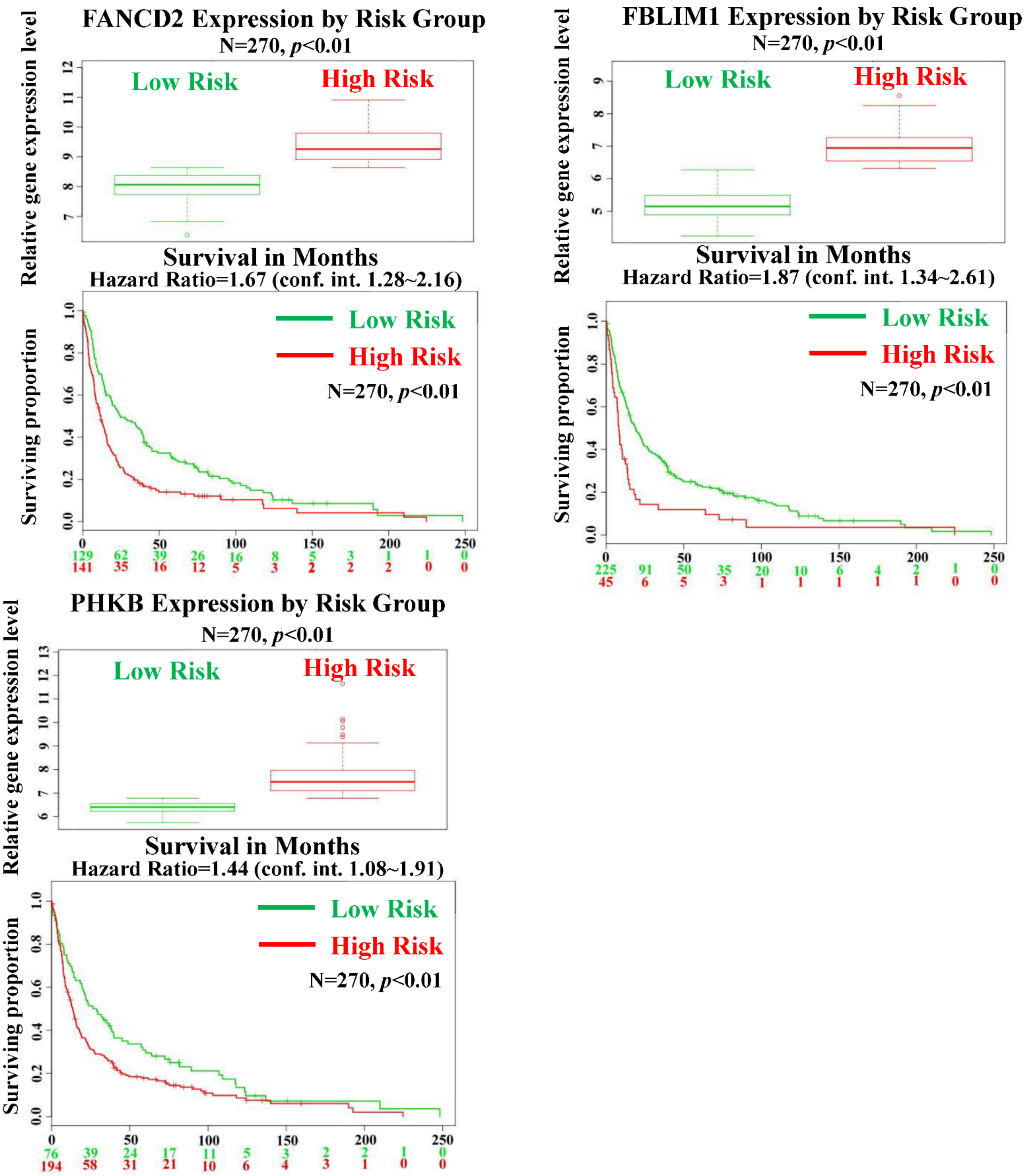

S1 Figure. Another seven target genes of miR-302b possessed characteristics similar to those of NFIA. Relative C3AR1, CLCN4, COL5A1, DHCR7, FANCD2, FBLIM1, and PHKB expression levels according to GBM patient risk group based on

GSE16011 profiling. Higher risk patients had higher gene expression, denoted by red symbols. Conversely, lower gene expression was associated with higher survival, denoted in green. Kaplan–Meier analysis of patient survival data from GSE16011 profiling. The *x*-axis values indicate the number of patients per group. The red curves indicate high-risk patients. The green curves indicate low-risk patients. The plus sign indicates censored observations. The *p* values were calculated using the log-rank test.
