# Supplementary material for: The microRNA-302b-inhibited insulin-like growth factor-binding protein 2 signaling pathway induces glioma cell apoptosis by targeting nuclear factor IA
Source: PLoS One. 2017 Mar 21;12(3):e0173890. doi: 10.1371/journal.pone.0173890 (PMC5360322; doi:10.1371/journal.pone.0173890)
Supplement: S2 Fig — (PDF) [file pone.0173890.s005.pdf]

S2 Fig

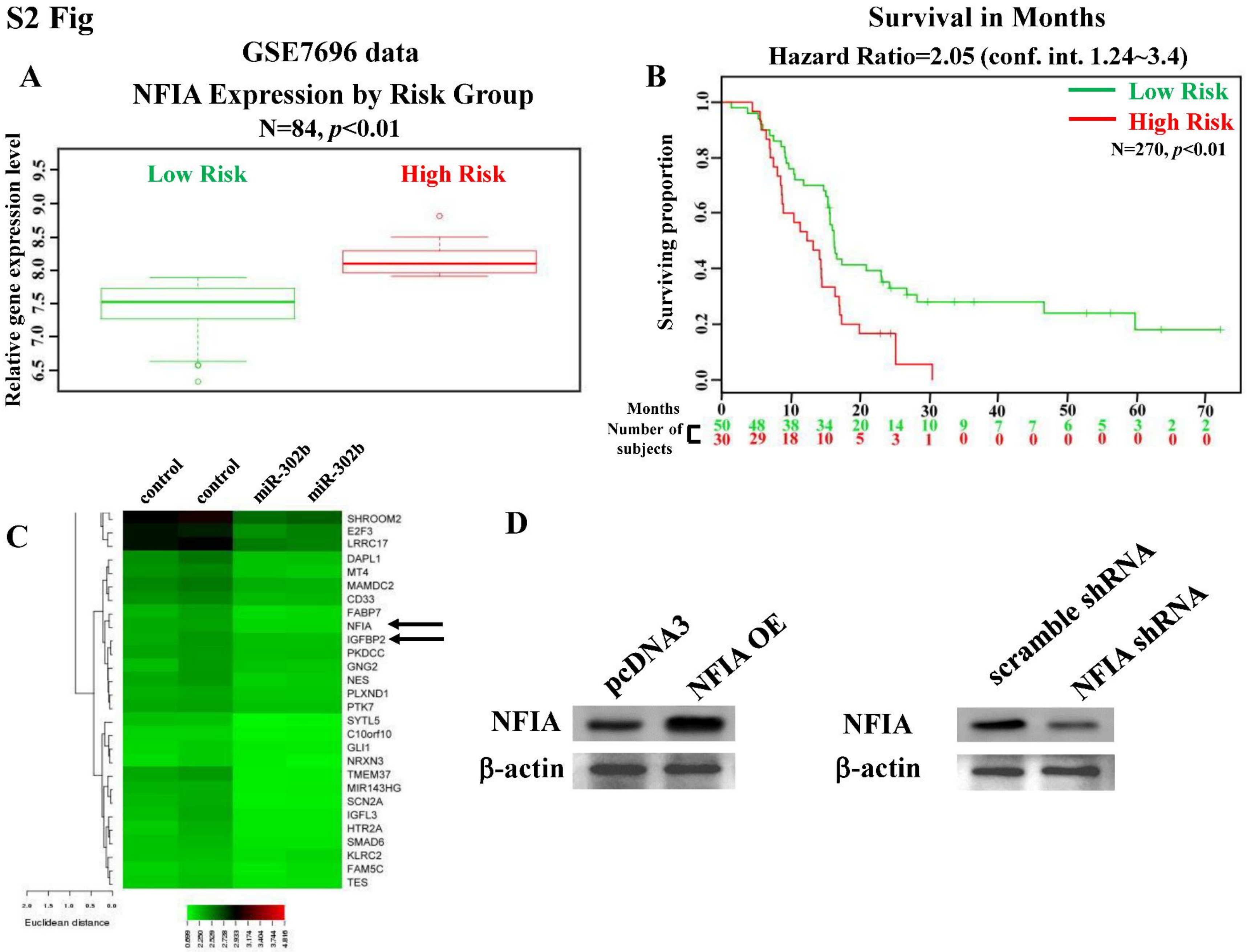
A
GSE7696 data
NFIA Expression by Risk Group
N=84, p<0.01
Relative gene expression level
Low Risk
High Risk
B
Survival in Months
Hazard Ratio=2.05 (conf. int. 1.24~3.4)
Surviving proportion
Low Risk
High Risk
N=270, p<0.01
Months
Number of subjects
C
control
control
miR-302b
miR-302b
SHROOM2
E2F3
LRRC17
DAPL1
MT4
MAMDC2
CD33
FABP7
NFIA
IGFBP2
PKDCC
GNG2
NES
PLXND1
PTK7
SYTL5
C10orf10
GLI1
NRXN3
TMEM37
MIR143HG
SCN2A
IGFL3
HTR2A
SMAD6
KLRC2
FAM5C
TES
Euclidean distance
D
pcDNA3
NFIA OE
NFIA
β-actin
scramble shRNA
NFIA shRNA
NFIA
β-actin

S2 Figure. Validation of the effects of NFIA on patient survival in another GEO dataset profiling. (A) Relative NFIA expression levels according to GBM patient risk group based on GSE7696 profiling. Higher risk patients had higher gene expression, denoted by red symbols. Conversely, lower gene expression was associated with higher survival, denoted in green. (B) Kaplan–Meier analysis of patient survival data from GSE7696 profiling. The *x*-axis values indicate the number of patients per group. The red curves indicate high-risk patients. The green curves indicate low-risk patients. The plus sign indicates censored observations. The *p* values were calculated using the log-rank test. (C) The heat map shows the relative NFIA and IGFBP2 levels. The figure of heat map was intercepted from group III of Fig. 1F. (D) Effects of overexpression and knockdown on NFIA levels. After overnight culture cells were transfected with 1 μg of NFIA OE plasmids, empty vectors, NFIA shRNA, and scrambled shRNA for 24 h, the NFIA protein levels were measured using immunoblotting.
