# Supplementary material for: The microRNA-302b-inhibited insulin-like growth factor-binding protein 2 signaling pathway induces glioma cell apoptosis by targeting nuclear factor IA
Source: PLoS One. 2017 Mar 21;12(3):e0173890. doi: 10.1371/journal.pone.0173890 (PMC5360322; doi:10.1371/journal.pone.0173890)
Supplement: S3 Fig — (PDF) [file pone.0173890.s006.pdf]

S3 Fig

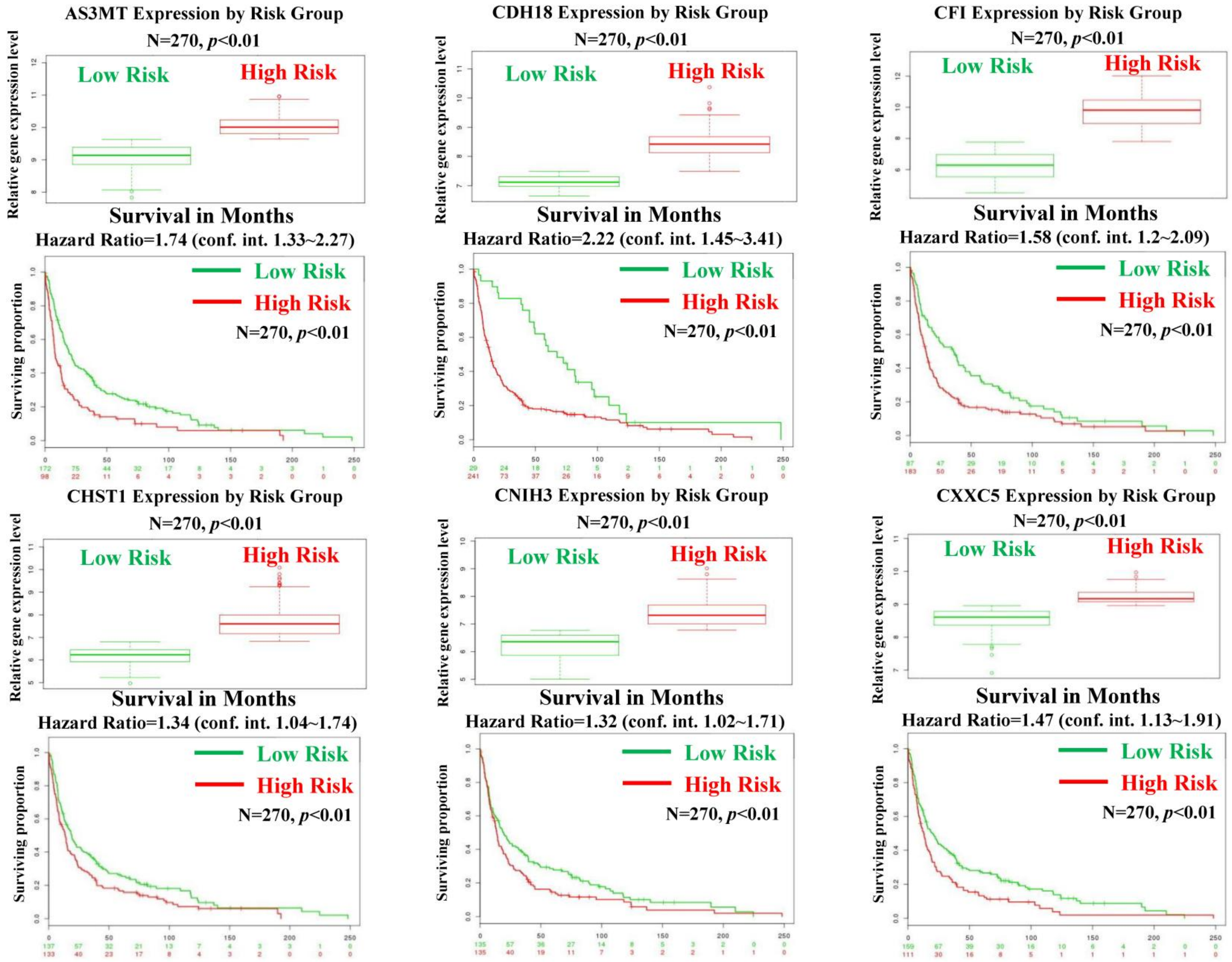
AS3MT Expression by Risk Group
N=270, p<0.01
Relative gene expression level
Low Risk
High Risk
Survival in Months
Hazard Ratio=1.74 (conf. int. 1.33~2.27)
Surviving proportion
Low Risk
High Risk
N=270, p<0.01
CDH18 Expression by Risk Group
N=270, p<0.01
Relative gene expression level
Low Risk
High Risk
Survival in Months
Hazard Ratio=2.22 (conf. int. 1.45~3.41)
Surviving proportion
Low Risk
High Risk
N=270, p<0.01
CFI Expression by Risk Group
N=270, p<0.01
Relative gene expression level
Low Risk
High Risk
Survival in Months
Hazard Ratio=1.58 (conf. int. 1.2~2.09)
Surviving proportion
Low Risk
High Risk
N=270, p<0.01
CHST1 Expression by Risk Group
N=270, p<0.01
Relative gene expression level
Low Risk
High Risk
Survival in Months
Hazard Ratio=1.34 (conf. int. 1.04~1.74)
Surviving proportion
Low Risk
High Risk
N=270, p<0.01
CNIH3 Expression by Risk Group
N=270, p<0.01
Relative gene expression level
Low Risk
High Risk
Survival in Months
Hazard Ratio=1.32 (conf. int. 1.02~1.71)
Surviving proportion
Low Risk
High Risk
N=270, p<0.01
CXXC5 Expression by Risk Group
N=270, p<0.01
Relative gene expression level
Low Risk
High Risk
Survival in Months
Hazard Ratio=1.47 (conf. int. 1.13~1.91)
Surviving proportion
Low Risk
High Risk
N=270, p<0.01

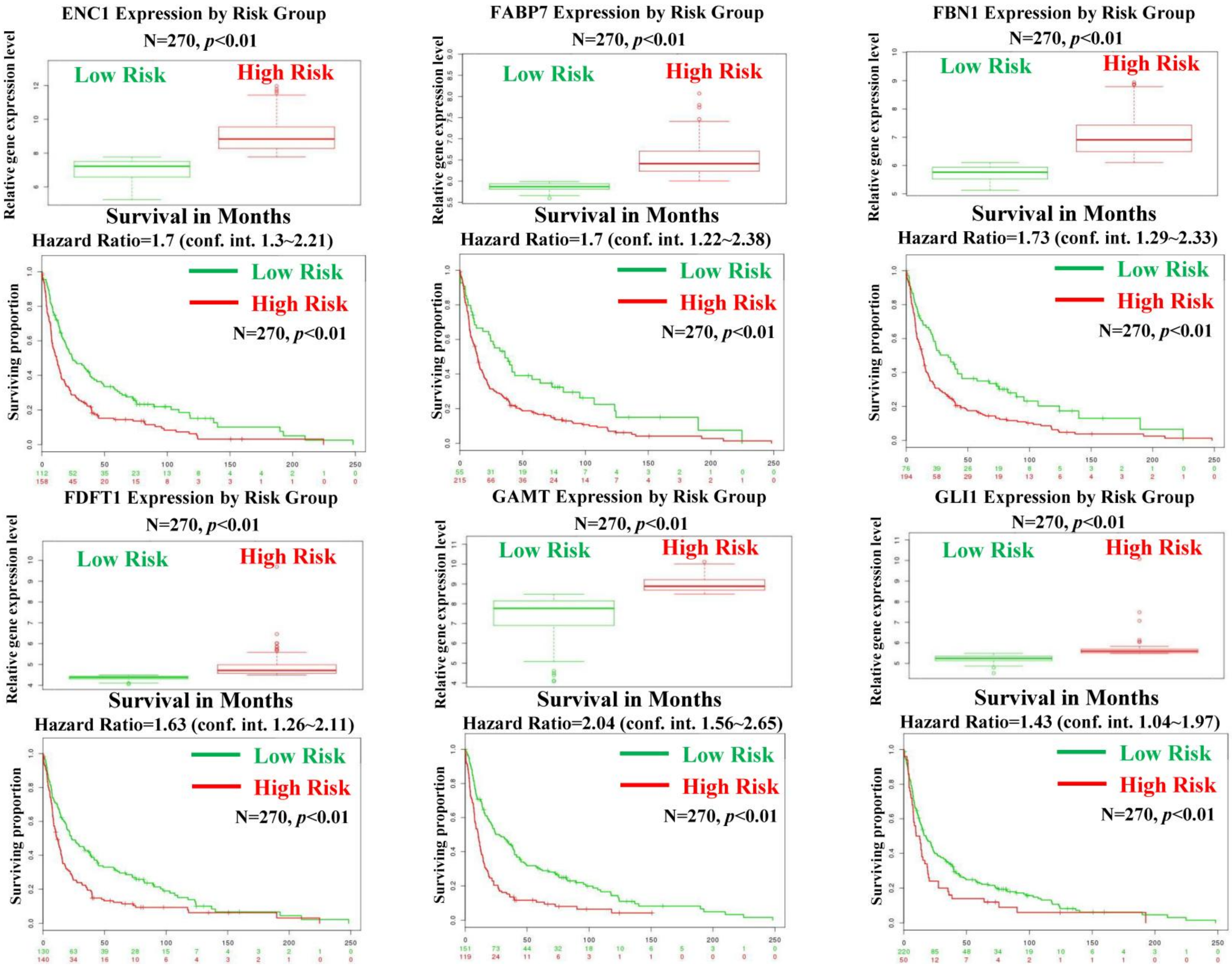

ENC1 Expression by Risk Group
N=270, p<0.01
Low Risk
High Risk
Relative gene expression level
Survival in Months
Hazard Ratio=1.7 (conf. int. 1.3~2.21)
Low Risk
High Risk
N=270, p<0.01
Surviving proportion
FABP7 Expression by Risk Group
N=270, p<0.01
Low Risk
High Risk
Relative gene expression level
Survival in Months
Hazard Ratio=1.7 (conf. int. 1.22~2.38)
Low Risk
High Risk
N=270, p<0.01
Surviving proportion
FBN1 Expression by Risk Group
N=270, p<0.01
Low Risk
High Risk
Relative gene expression level
Survival in Months
Hazard Ratio=1.73 (conf. int. 1.29~2.33)
Low Risk
High Risk
N=270, p<0.01
Surviving proportion
FDFT1 Expression by Risk Group
N=270, p<0.01
Low Risk
High Risk
Relative gene expression level
Survival in Months
Hazard Ratio=1.63 (conf. int. 1.26~2.11)
Low Risk
High Risk
N=270, p<0.01
Surviving proportion
GAMT Expression by Risk Group
N=270, p<0.01
Low Risk
High Risk
Relative gene expression level
Survival in Months
Hazard Ratio=2.04 (conf. int. 1.56~2.65)
Low Risk
High Risk
N=270, p<0.01
Surviving proportion
GLI1 Expression by Risk Group
N=270, p<0.01
Low Risk
High Risk
Relative gene expression level
Survival in Months
Hazard Ratio=1.43 (conf. int. 1.04~1.97)
Low Risk
High Risk
N=270, p<0.01
Surviving proportion

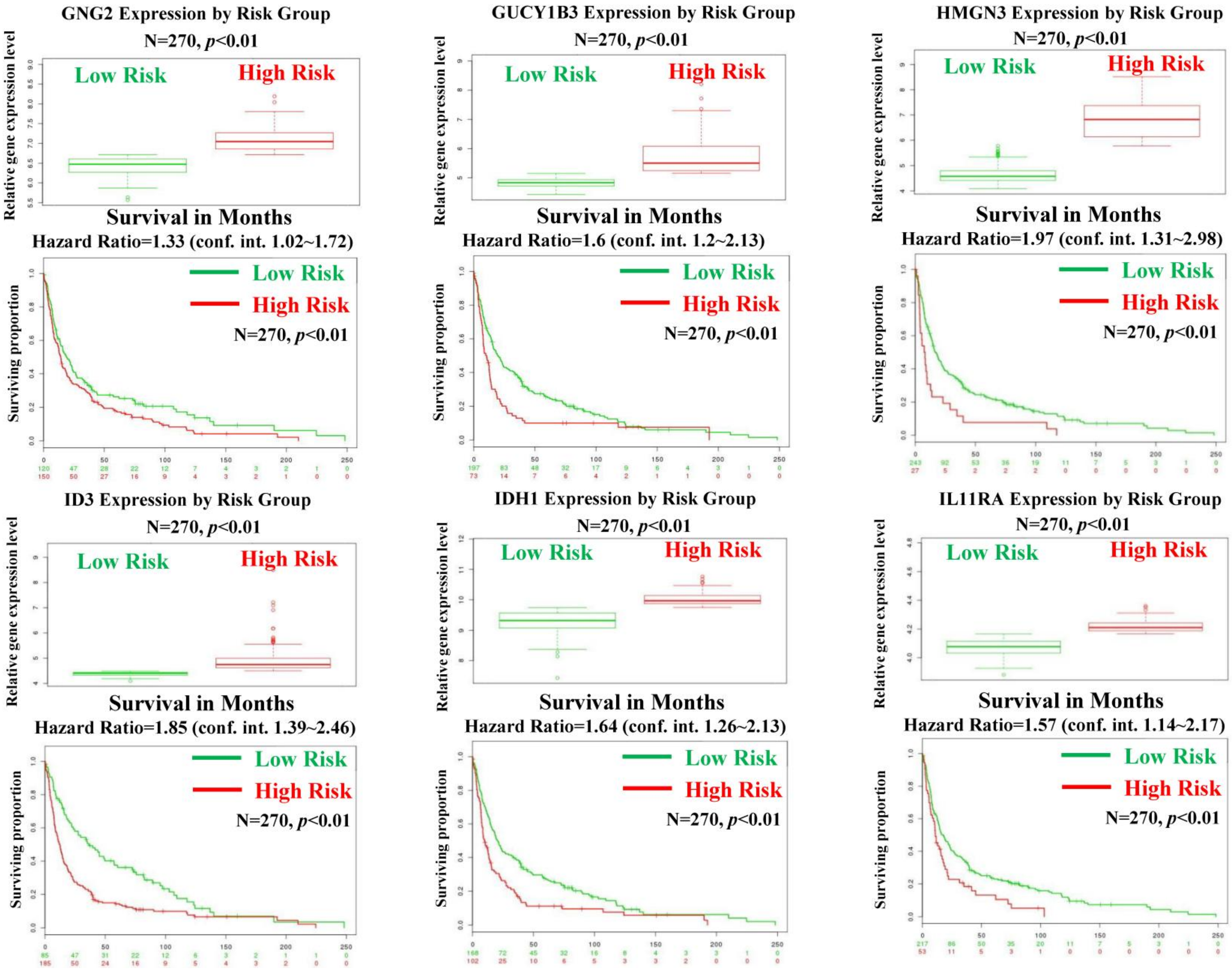

GNG2 Expression by Risk Group
N=270, p<0.01
Low Risk
High Risk
Relative gene expression level
Survival in Months
Hazard Ratio=1.33 (conf. int. 1.02~1.72)
Low Risk
High Risk
N=270, p<0.01
Surviving proportion
GUCY1B3 Expression by Risk Group
N=270, p<0.01
Low Risk
High Risk
Relative gene expression level
Survival in Months
Hazard Ratio=1.6 (conf. int. 1.2~2.13)
Low Risk
High Risk
N=270, p<0.01
Surviving proportion
HMGN3 Expression by Risk Group
N=270, p<0.01
Low Risk
High Risk
Relative gene expression level
Survival in Months
Hazard Ratio=1.97 (conf. int. 1.31~2.98)
Low Risk
High Risk
N=270, p<0.01
Surviving proportion
ID3 Expression by Risk Group
N=270, p<0.01
Low Risk
High Risk
Relative gene expression level
Survival in Months
Hazard Ratio=1.85 (conf. int. 1.39~2.46)
Low Risk
High Risk
N=270, p<0.01
Surviving proportion
IDH1 Expression by Risk Group
N=270, p<0.01
Low Risk
High Risk
Relative gene expression level
Survival in Months
Hazard Ratio=1.64 (conf. int. 1.26~2.13)
Low Risk
High Risk
N=270, p<0.01
Surviving proportion
IL11RA Expression by Risk Group
N=270, p<0.01
Low Risk
High Risk
Relative gene expression level
Survival in Months
Hazard Ratio=1.57 (conf. int. 1.14~2.17)
Low Risk
High Risk
N=270, p<0.01
Surviving proportion

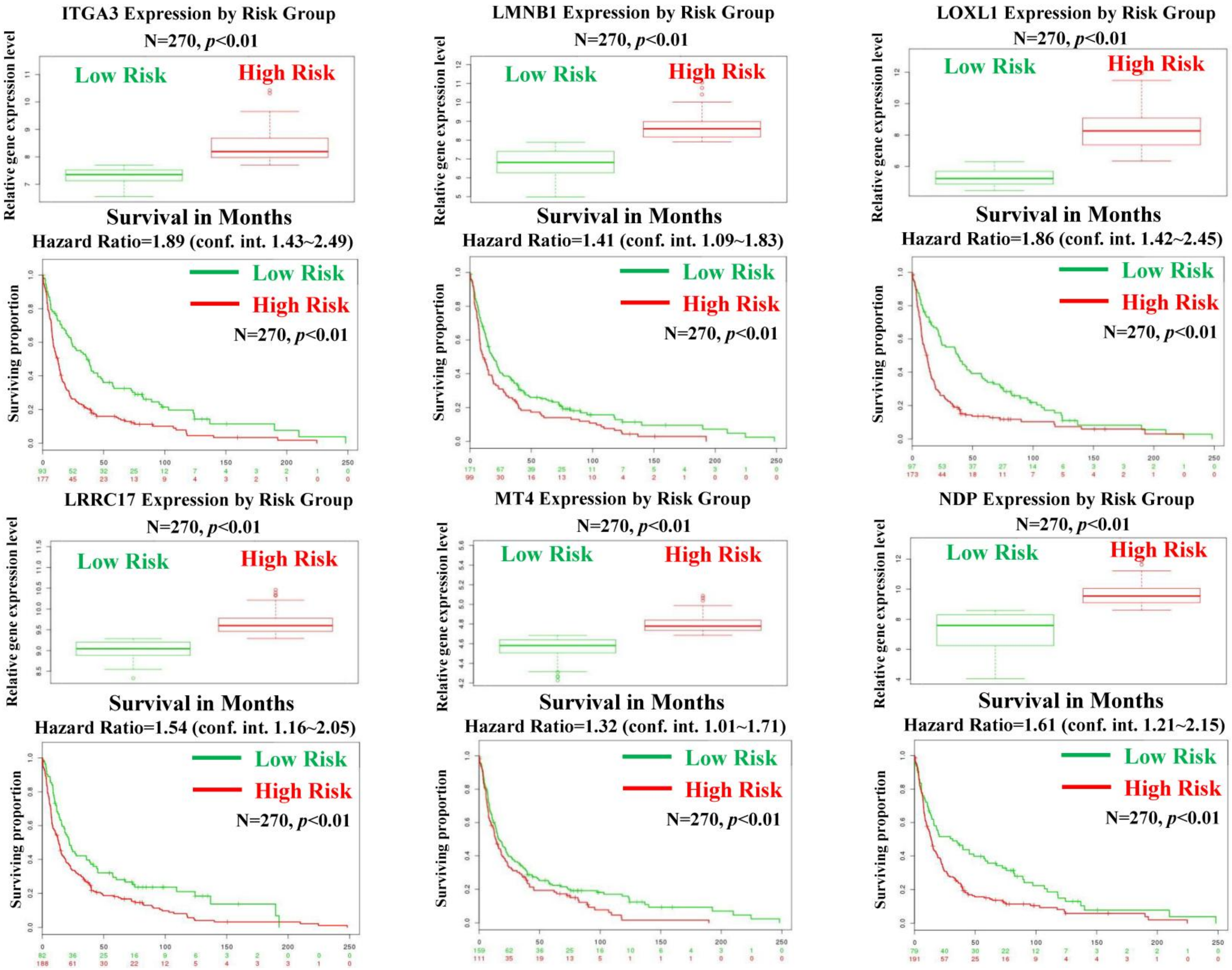
ITGA3 Expression by Risk Group
N=270, p<0.01
Low Risk
High Risk
Relative gene expression level
Survival in Months
Hazard Ratio=1.89 (conf. int. 1.43~2.49)
Low Risk
High Risk
N=270, p<0.01
Surviving proportion
LMNB1 Expression by Risk Group
N=270, p<0.01
Low Risk
High Risk
Relative gene expression level
Survival in Months
Hazard Ratio=1.41 (conf. int. 1.09~1.83)
Low Risk
High Risk
N=270, p<0.01
Surviving proportion
LOXL1 Expression by Risk Group
N=270, p<0.01
Low Risk
High Risk
Relative gene expression level
Survival in Months
Hazard Ratio=1.86 (conf. int. 1.42~2.45)
Low Risk
High Risk
N=270, p<0.01
Surviving proportion
LRRC17 Expression by Risk Group
N=270, p<0.01
Low Risk
High Risk
Relative gene expression level
Survival in Months
Hazard Ratio=1.54 (conf. int. 1.16~2.05)
Low Risk
High Risk
N=270, p<0.01
Surviving proportion
MT4 Expression by Risk Group
N=270, p<0.01
Low Risk
High Risk
Relative gene expression level
Survival in Months
Hazard Ratio=1.32 (conf. int. 1.01~1.71)
Low Risk
High Risk
N=270, p<0.01
Surviving proportion
NDP Expression by Risk Group
N=270, p<0.01
Low Risk
High Risk
Relative gene expression level
Survival in Months
Hazard Ratio=1.61 (conf. int. 1.21~2.15)
Low Risk
High Risk
N=270, p<0.01
Surviving proportion

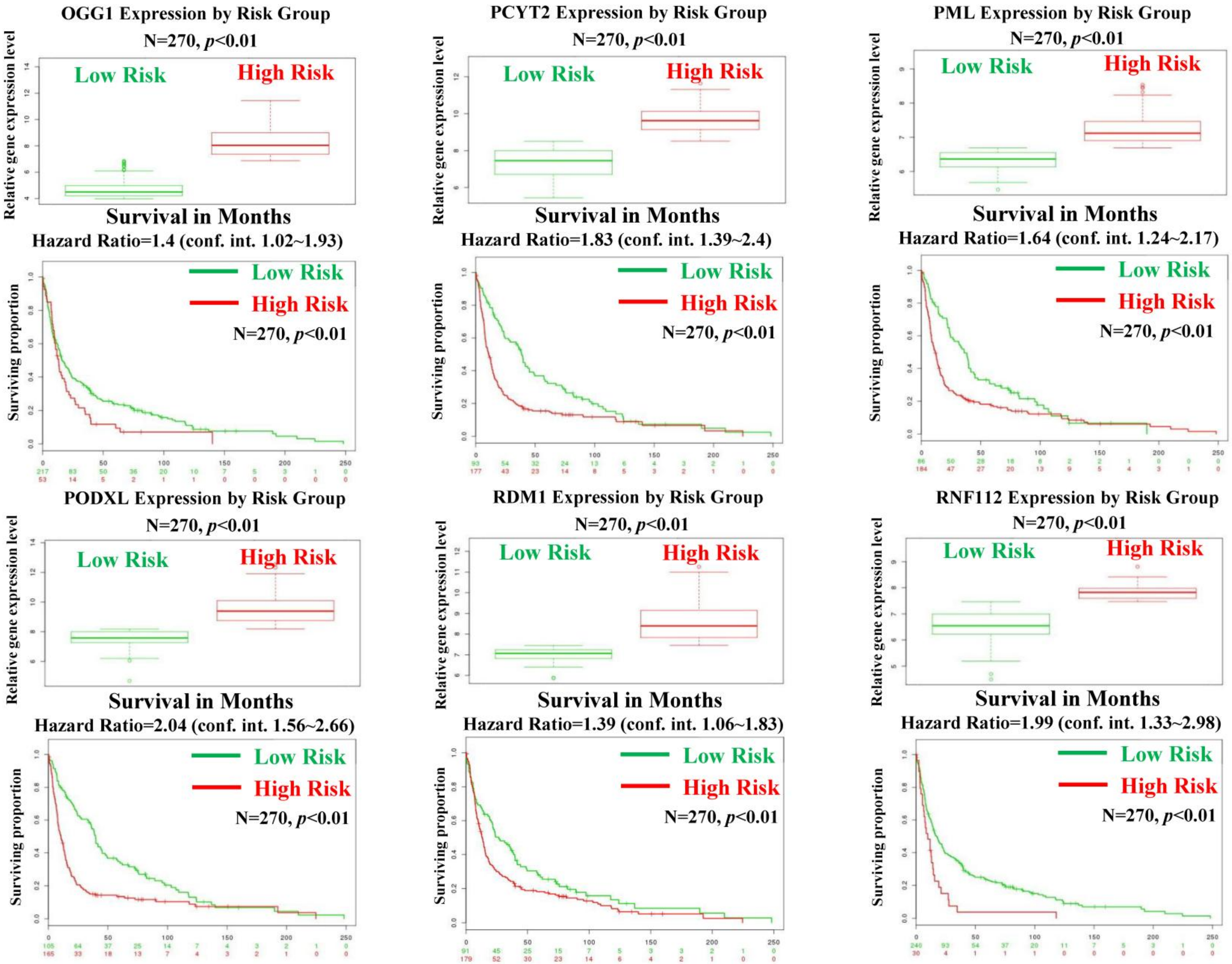

OGG1 Expression by Risk Group
N=270, p<0.01
Low Risk
High Risk
Relative gene expression level
Survival in Months
Hazard Ratio=1.4 (conf. int. 1.02~1.93)
Low Risk
High Risk
N=270, p<0.01
Surviving proportion
PCYT2 Expression by Risk Group
N=270, p<0.01
Low Risk
High Risk
Relative gene expression level
Survival in Months
Hazard Ratio=1.83 (conf. int. 1.39~2.4)
Low Risk
High Risk
N=270, p<0.01
Surviving proportion
PML Expression by Risk Group
N=270, p<0.01
Low Risk
High Risk
Relative gene expression level
Survival in Months
Hazard Ratio=1.64 (conf. int. 1.24~2.17)
Low Risk
High Risk
N=270, p<0.01
Surviving proportion
PODXL Expression by Risk Group
N=270, p<0.01
Low Risk
High Risk
Relative gene expression level
Survival in Months
Hazard Ratio=2.04 (conf. int. 1.56~2.66)
Low Risk
High Risk
N=270, p<0.01
Surviving proportion
RDM1 Expression by Risk Group
N=270, p<0.01
Low Risk
High Risk
Relative gene expression level
Survival in Months
Hazard Ratio=1.39 (conf. int. 1.06~1.83)
Low Risk
High Risk
N=270, p<0.01
Surviving proportion
RNF112 Expression by Risk Group
N=270, p<0.01
Low Risk
High Risk
Relative gene expression level
Survival in Months
Hazard Ratio=1.99 (conf. int. 1.33~2.98)
Low Risk
High Risk
N=270, p<0.01
Surviving proportion

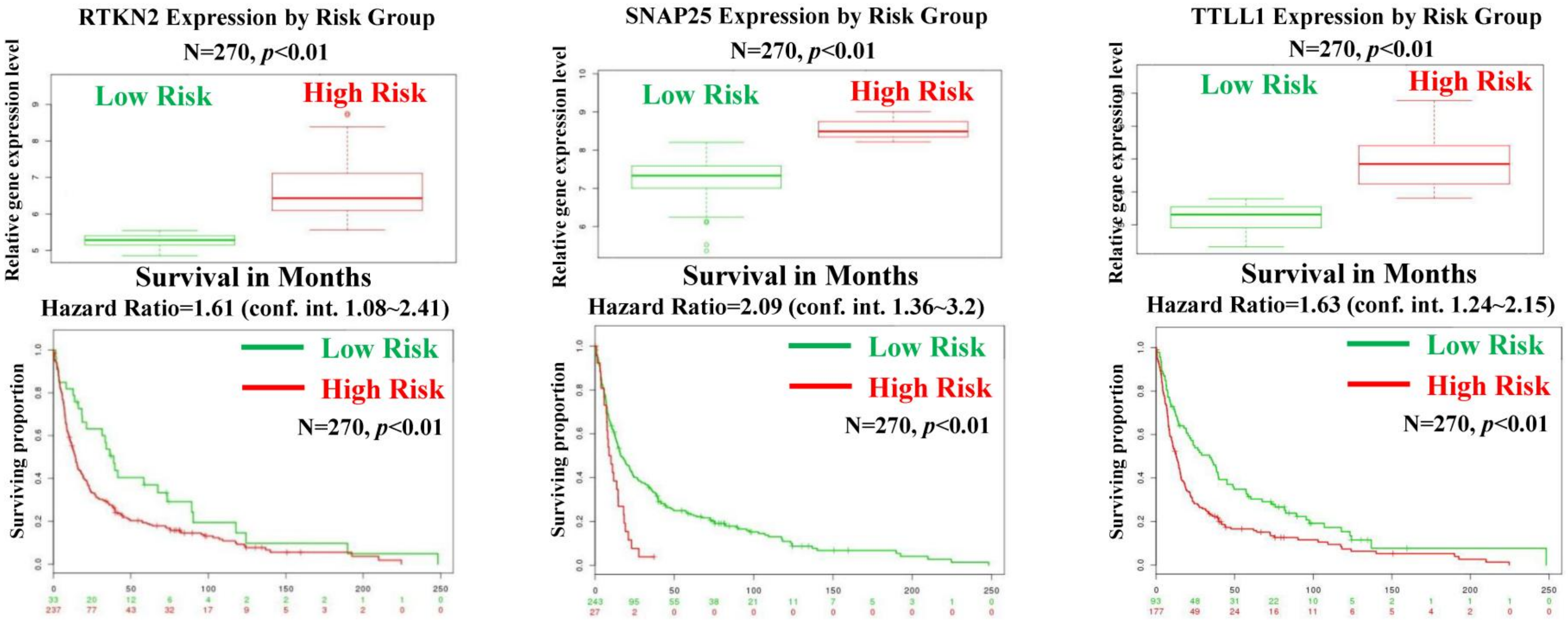

S3 Figure. Another 33 nondirect target genes of miR-302b possessed characteristics similar to those of NFIA. Relative AS3MT, CDH18, CFI, CHST1, CNIH3, CXXC5, ENC1, FABP7, FBN1, FDFT1, GAMT, GLI1, GNG2, GUCY1B3, HMGN3, ID3, IDH1, IL11RA, ITGA3, LMNB1, LOXL1, LRRC17, MT4, NDP, OGG1, PCYT2, PML, PODXL, RDM1, RNF112, RTKN2, SNAP25, and TTLL1 expression levels according to GBM patient risk group based on GSE16011 profiling. Higher risk individuals had higher gene expression, denoted by red symbols. Conversely, lower gene expression was associated with higher survival, denoted in green. Kaplan–Meier analysis of patient survival data from GSE16011 profiling. The *x*-axis values indicate the number of patients per group. The red curves indicate high-risk patients. The green curves indicate low-risk patients. The plus sign indicates censored observations. The *p* values were calculated using the log-rank test.
